# CDC42 governs normal oviduct multiciliogenesis through activating AKT to ensure timely embryo transport

**DOI:** 10.1038/s41419-022-05184-y

**Published:** 2022-09-02

**Authors:** Ruiwei Jiang, Xiaofang Tang, Jiale Pan, Gaizhen Li, Ningjie Yang, Yedong Tang, Shilei Bi, Han Cai, Qionghua Chen, Dunjin Chen, Haibin Wang, Shuangbo Kong

**Affiliations:** 1 Fujian Provincial Key Laboratory of Reproductive Health Research, Department of Obstetrics and Gynecology, The First Affiliated Hospital of Xiamen University, School of Medicine, Xiamen University, 361102 Xiamen, Fujian China; 2 Center for Reproductive Medicine and Obstetrics and Gynecology, Nanjing Drum Tower Hospital, Nanjing University Medical School, 210093 Nanjing, Jiangsu China; 3 Department of Obstetrics and Gynecology, Key Laboratory for Major Obstetric Diseases of Guangdong Province, The Third Affiliated Hospital of Guangzhou Medical University, 510150 Guangzhou, Guangdong China

**Keywords:** Mechanisms of disease, Infertility

## Abstract

Ciliated and secretory cells are two major cell types that comprise the oviduct epithelia. Accumulating evidences support a role of oviductal multiciliated epithelia for embryo transport, however the mechanisms underlying this specialized cell type differentiation remain elusive. Here, we report that CDC42 depletion in oviduct epithelia hampers the morphogenesis of multiciliated cell, and results in embryo retention, leading to early pregnancy failure. Utilizing the oviduct organoid model, we further observed that CDC42 guides secretory cells transition into multiciliated cells independent of its GTPase activity and the well-known Notch pathway. Further exploration uncovered the AKT as a novel indispensable regulator for multiciliated cells differentiation, whose activity was maintained by CDC42 through interacting with the p110β. Consistently, re-activating AKT partially incites multiciliated cells differentiation in *Cdc42* knockout oviductal organoids. Finally, low levels of CDC42 and phospho-AKT with reduced multiciliated cells in the oviduct are observed in women with ectopic pregnancy. Collectively, we provide previously unappreciated evidence that CDC42-AKT signaling is a critical determinant for morphogenesis of oviduct multiciliated cell, which possesses the clinical application in understanding the pathology of ectopic pregnancy and facilitating the development of prevention strategies.

## Introduction

Oviducts (analogous to the fallopian tube in humans) are a pair of narrow tubular structure that serves as a conduit between the ovary and the uterus to support fertilization, preimplantation embryonic development and timely transport of embryo [1]. Fertilization occurs in the ampulla, then developing embryo pass the isthmus and uterotubal junction to enter the uterine cavity for implantation in the endometrium. Embryo transport is believed to be achieved by complex interaction of smooth muscle contraction, cilia beating, and flow of tubal fluid in the oviduct [2, 3]. When a preimplantation embryo does not properly traverse the fallopian tube, for example, due to an obstruction or constriction in the luminal space, the incidence of infertility, and ectopic pregnancy in women will significantly increase [4].

The oviductal mucosal epithelium consists of two major cell types: multiciliated cells and nonciliated secretory cells. The distribution of these two cell types is not equal along the whole oviduct, with the relatively high proportion of multiciliated cells in the infundibulum and ampulla compared with a low proportion in the isthmus and uterotubal junction [5]. Multiciliated cells are thought to be important for embryo transport since they bear the motile cilium structure for beating to guide the flow direction. Evidence is accumulating for the important and possibly preeminent role of multiciliated cells in the isthmus and ampulla during the embryo transport process, although the relative contributions of smooth muscle contraction remain debatable [2, 6–8]. Despite the importance of oviductal epithelial cell dynamics in homeostasis and disease, the mechanisms underlying the cell fate determination of multiciliated cell remain indescribable. Electron microscopy shows that occasional epithelial cells in fallopian tube possess the features of both ciliated and secretory cells, suggesting that these two cell types might be interchangeable [9]. Using the in vitro cultured organoid derived from human fallopian tube epithelia, it was demonstrated that bipotent tubal epithelial stem cells can give rise to both ciliated and secretory cells [10]. In vivo lineage tracing also reveals that PAX8 positive secretory cells act as oviductal epithelial progenitors, which expand over time to repopulate the whole oviductal epithelium and give rise to FOXJ1 positive ciliated cells [11]. Recent single-cell transcriptomic profiling of human fallopian tube specimens provides a molecular trajectory whereby secretory cells differentiate into ciliated cells via a RUNX3^high^ intermediate, while the data from mice identified transcriptionally distinct populations of secretory and multiciliated cells restricted to the distal and proximal regions of the oviduct [12, 13]. The process of multiciliated cells differentiation, or multiciliogenesis, requires the activation of a unique transcriptional program that specifies cell fate and allows the massive amplification of centrioles [14]. Seminal work has demonstrated that Inhibition of Notch causes the trans-differentiation of secretory cells into multiciliated cells in multiple tissues, including the airway and oviduct [10, 15–17]. However, the underlying mechanism besides Notch signaling regulating multiciliated cell differentiation remains elusive.

CDC42, a member of the small Rho GTPase family, regulates multi-cellular functions, including actin cytoskeletal dynamics, membrane trafficking, transcription, and cell cycle progression [18, 19]. CDC42 is an essential regulator of epithelial morphogenesis, through coordination of apical membrane morphogenesis, lumen formation, and junction maturation. Typically, the apical membrane of luminal epithelium consists of tightly structured microvilli or motile cilia in tissue dependent context [20]. Interestingly, previous studies have found that CDC42 regulates the primary ciliogenesis of kidney tubular epithelial cells [21, 22]. However, the role of CDC42 in oviduct multiciliogenesis has not been thoroughly described until now. Here we report that conditional knockout of *Cdc42* in the oviduct epithelial cells at the isthmus region causes decreased number of multiciliated cells, contributing to retention of embryos in the isthmus, eventually leading to early pregnancy failure. CDC42 directs the cell fate transition of oviduct secretory cells into multiciliated cells independent on its GTPase activity or Notch signaling, and further exploration uncovered the AKT as an indispensable regulator, whose activity was regulated by CDC42 through interacting with the P110β. Finally, the decreased expression of CDC42 and phospho-AKT with reduced number of ciliated cells are also evidenced in the fallopian tube of women with ectopic pregnancy, indicating the significance of CDC42-PI3K-AKT signaling axis in morphogenesis of multiciliated cells and ectopic pregnancy, which will provide insightful information for the development of new therapeutic strategies.

## Results

### Loss of CDC42 in the oviduct leads to defective embryo transport

**Fig. 1:**
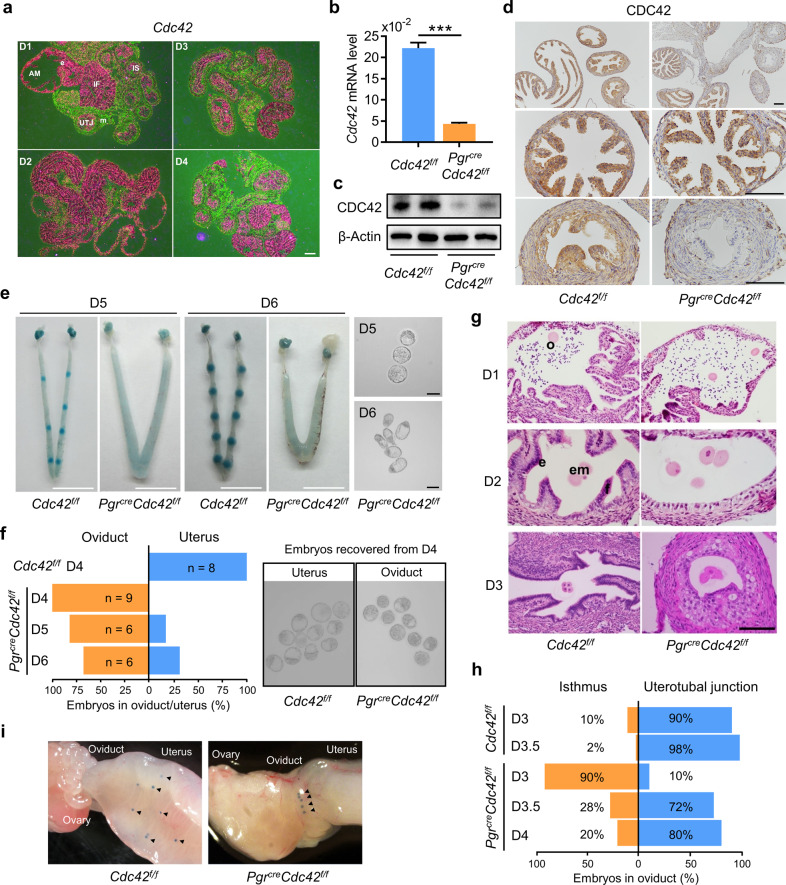
Loss of CDC42 in the oviduct leads to defective embryo transport. **a** In situ hybridization of *Cdc42* mRNA in D1-4 oviducts. **b** RT-qPCR analysis of *Cdc42* mRNA and **c** Western blot analysis of CDC42 protein in *Cdc42*^*f/f*^ and *Pgr*^*cre*^*Cdc42*^*f/f*^ mouse oviducts. **d** Immunostaining showed that CDC42 was lost in the isthmus, but not in the ampulla of *Pgr*^*cre*^*Cdc42*^*f/f*^ mouse oviducts. **e** *Pgr*^*cre*^*Cdc42*^*f/f*^ mice exhibit implantation failure recovered with small numbers of morphologically normal blastocysts upon flushing the uterine horn on day 5 and 6 of pregnancy. **f** Percentage of embryos recovered from oviducts or uteri. Recovered blastocysts from *Cdc42*^*f/f*^ mouse uteri and *Pgr*^*cre*^*Cdc42*^*f/f*^ mouse oviducts on day 4. **g** Oocytes in the ampulla on day 1 and embryos in the isthmus on day 2 of both *Cdc42*^*f/f*^ and *Pgr*^*cre*^*Cdc42*^*f/f*^ mice. Embryos enter in the uterobutal junction of *Cdc42*^*f/f*^ mice, while retained in the isthmus of *Pgr*^*cre*^*Cdc42*^*f/f*^ mice on day 3. **h** Percentage of embryos recovered from the isthmus and uterotubal junction. **i** Affi-Gel Blue Gel beads transferred into the oviduct umbrella of D1 pseudopregnant mice enter in the uterus of *Cdc42*^*f/f*^ mice, while retained in the isthmus of *Pgr*^*cre*^*Cdc42*^*f/f*^ mice on day 4. Arrowheads, sites of transferred gel beads. IF infundibulum, AM ampulla, IS isthmus, e epithelium, em embryo, o oocyte. Black scale bars, 100 μm. While scale bars, 1 cm.

To detect the expression and location of CDC42 in mouse oviducts from days 1 to 4 of pregnancy (D1–4), we performed in situ hybridization and immunohistochemistry (IHC) and demonstrated that CDC42 was mainly located in the epithelial cells of the infundibulum, ampulla, isthmus, and uterotubal junction (Fig. 1a and Supplementary Fig. 1). A progesterone receptor Cre line (*Pgr*^*Cre*^) was bred with *Cdc42*^*f/f*^ mice to create conditional knockout of *Cdc42* in reproductive tract (termed *Pgr*^*Cre*^*Cdc42*^*f/f*^ mice) beginning around 6 days after birth [23]. qPCR and western blot assays showed that CDC42 was reduced in the *Pgr*^*Cre*^*Cdc42*^*f/f*^ mice compared with the *Cdc42*^*f/f*^ mice, while IHC data further confirmed that CDC42 was absent only in the isthmus region of *Pgr*^*Cre*^*Cdc42*^*f/f*^ mice (Fig. 1b–d). There were no pups born in *Pgr*^*Cre*^*Cdc42*^*f/f*^ females after a 6-month breading, and we found that none of the *Pgr*^*Cre*^*Cdc42*^*f/f*^ female showed implantation sites as visible by blue dye reaction, albeit morphologically normal blastocysts can be recovered by flushing the *Pgr*^*Cre*^*Cdc42*^*f/f*^ uteri on D5 and D6 (Fig. 1e). The potential ovarian defects were excluded by the fact of similar ovarian steroid hormones estradiol-17β (E2) and progesterone (P4), as well as normal expression of P450- Side Chain Cleavage Enzyme (scc) in *Cdc42*^*f/f*^ and *Pgr*^*Cre*^*Cdc42*^*f/f*^ mice on D4 (Supplementary Fig. 2).

The relative less embryos retrieved from Cdc42 deficient mice led us to postulate that the embryo transport through the oviduct may be impeded. We found that all the blastocysts had entered the uterus on D4 in the control *Cdc42*^*f/f*^ mice. However, in *Pgr*^*Cre*^*Cdc42*^*f/f*^ mice, all the embryos were retained in the oviduct on D4, with only 15% and 28% embryos entered the uteri on D5 and D6, respectively (Fig. 1f). Although embryos were observed in infundibulum, ampulla, and isthmus in *Cdc42*^*f/f*^ and *Pgr*^*Cre*^*Cdc42*^*f/f*^ mouse oviducts from D1 to D3 (Fig. 1g), there was only 10% embryos passed the isthmus on D3 in *Pgr*^*Cre*^*Cdc42*^*f/f*^ mice compared with 90% in *Cdc42*^*f/f*^ mice. There were 28% embryos retained in isthmus in *Pgr*^*Cre*^*Cdc42*^*f/f*^ mice compared with none in D3.5 *Cdc42*^*f/f*^ mice. Even on D4, there were still 20% embryos restricted in isthmus of *Pgr*^*Cre*^*Cdc42*^*f/f*^ mice (Fig. 1h). To confirm the autonomous defect of knockout oviduct, we transplanted the Affi-Gel Blue Gel beads into the ampulla of pseudo-pregnant mice to exclude the potential embryonic factor attributed to the hostile environment. Similarly, the blue beads all entered the uterine cavity in the pseudo-pregnant *Cdc42*^*f/f*^ mice on D4, but were retained in the oviductal isthmus of pseudo-pregnant *Pgr*^*Cre*^*Cdc42*^*f/f*^ mice on D4 (Fig. 1i). The above results indicated that knockout of *Cdc42* in the epithelial cells of the oviductal isthmus led to defective embryo transport, resulting in blastocysts retention in oviduct, and contributing to the early pregnancy failure in *Pgr*^*Cre*^*Cdc42*^*f/f*^ mice.

### CDC42 deficient mice lacks multiciliated cells in the oviduct

**Fig. 2:**
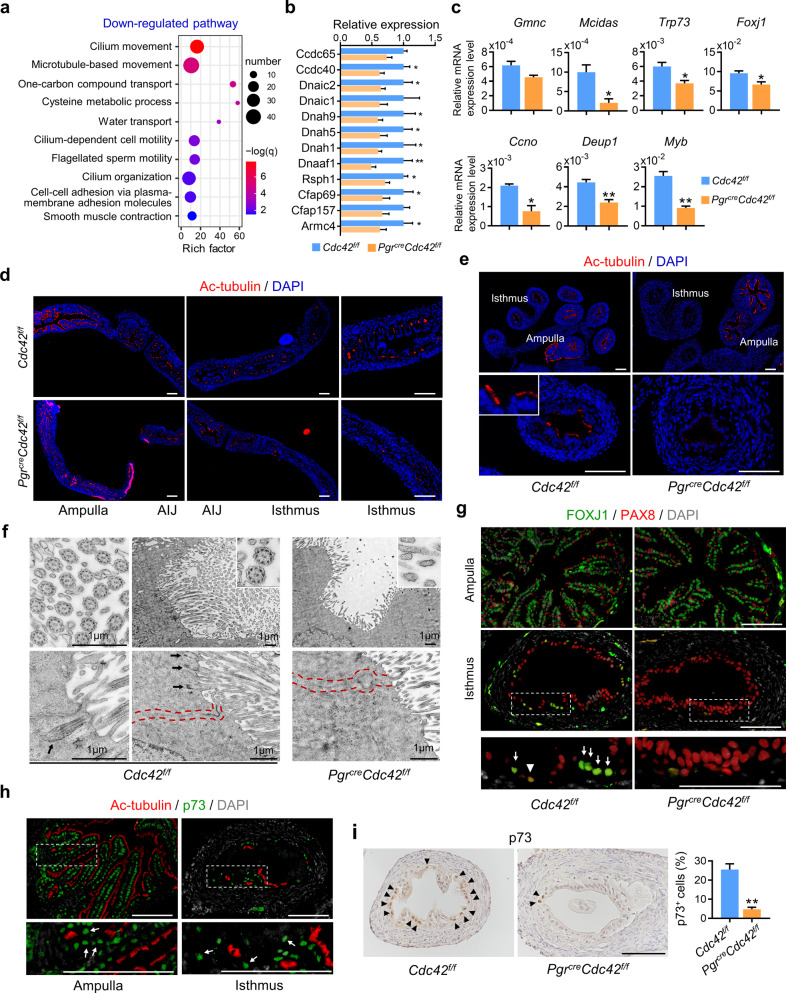
CDC42 deficient mice lacks multiciliated cells in the oviduct. **a** Go enrichment analysis of down-regulated genes in the *Pgr*^*cre*^*Cdc42*^*f/f*^ mouse oviducts on day 2. **b** Relative expression levels of ciliary movement related genes were decreased in the *Pgr*^*cre*^*Cdc42*^*f/f*^ mouse oviducts on day 2. **c** RT-qPCR analysis of genes involved in transcription regulation of multiciliogenesis, Gmnc, Mcidas, Trp73, and Foxj1, and genes regulating basal body amplification, *Ccno*, *Deup*, and *Myb* between the *Cdc42*^*f/f*^ and *Pgr*^*cre*^*Cdc42*^*f/f*^ mouse oviducts on day 2. **d** Immunostaining of acetylated tubulin (Ac-tubulin) in the longitudinal section of oviducts to show cilia distribution in the ampulla, ampulla-isthmus junction (AIJ) and isthmus of *Cdc42*^*f/f*^ and *Pgr*^*cre*^*Cdc42*^*f/f*^ mice on day 2. **e** Immunostaining of Ac-tubulin in the cross section of ampulla and isthmus of the *Cdc42*^*f/f*^ and *Pgr*^*cre*^*Cdc42*^*f/f*^ mouse oviducts in diestrus. **f** Transmission electron microscopy showed the “9 + 2” motile cilia and positioned basal bodies (arrows) in the isthmus of *Cdc42*^*f/f*^ mouse oviducts, while only microvillus in the isthmus of *Pgr*^*cre*^*Cdc42*^*f/f*^ mouse oviducts on D2. **g** Dual immunostaining showed the FOXJ1^+^ ciliated cells (arrows) and PAX8^+^ secretory cells in the isthmus of *Cdc42*^*f/f*^ and *Pgr*^*cre*^*Cdc42*^*f/f*^ mouse oviducts, noting the FOXJ1 and PAX8 dual-positive cells (arrowheads) in the *Cdc42*^*f/f*^ mice in diestrus. **h** Dual immunostaining of Ac-tubulin and p73 in the ampulla and isthmus of *Cdc42*^*f/f*^ mice, noting the p73^+^ Ac-tubulin^-^ cells which have committed to ciliated cell fate (Arrows) in diestrus. **i** Immunostaining of p73 in the isthmus showed reduced number of p73^+^ cells (arrowheads) in the *Pgr*^*cre*^*Cdc42*^*f/f*^ mice in diestrus. Scale bars, 100 μm. Mean ± SD. *P < 0.05, **P < 0.01, Student’s *t* test.

To further determine the role of CDC42 in oviduct, RNA-seq analysis was applied in the *Cdc42*^*f/f*^ and *Pgr*^*Cre*^*Cdc42*^*f/f*^ oviduct on D2. There were 915 genes up-regulated and 893 genes down-regulated in the *Pgr*^*Cre*^*Cdc42*^*f/f*^ oviduct compared with the control. Gene ontology (GO) enrichment analysis of Down-regulated genes in the *Pgr*^*Cre*^*Cdc42*^*f/f*^ mouse oviducts were abundantly enriched in the cilia movement and cilia assembly pathways, as well as cell-cell adhesion and smooth muscle contraction (Fig. 2a). Both RNA-Seq and qPCR evidenced that the expression of ciliary movement related genes *Cfap69*, *Rsph1*, *Dnaaf1*, *Dnah1*, *Dnaic2*, and *Ccdc40*, the key multiciliogenesis transcription factors *Mcidas*, *Trp73* and *Foxj1*, and basal body amplification regulators *Ccno*, *Deup1*, and *Myb* were significantly reduced in *Pgr*^*Cre*^*Cdc42*^*f/f*^ oviduct (Fig. 2b, c). The expression of acetylated tubulin (Ac-tubulin), a marker of ciliated cells, revealed that ciliated cells were most abundant in the ampulla followed by ampulla-Isthmus junction (AIJ) and isthmus in *Cdc42*^*f/f*^ mouse oviducts. While the ciliated cells dramatically decreased in *Pgr*^*Cre*^*Cdc42*^*f/f*^ isthmus, which recapitulated the recombination activity of *Pgr*^*Cre*^ in oviductal isthmus (Fig. 2d, e). Similar reduction was also observed in D3 *Pgr*^*Cre*^*Cdc42*^*f/f*^ mouse oviducts (Supplementary Fig. 3a). Using transmission electron microscopy, we found that the multiciliated cells in the control oviductal isthmus presented a classical motile ciliated cell structure (9 + 2) and normal basal body docking. However, normal motile cilia and basal bodies were absent in the oviductal isthmus of *Pgr*^*Cre*^*Cdc42*^*f/f*^ mice, but with dense microvilli (Fig. 2f). Immunofluorescence staining with anti-pericentrin, a marker for basal body, unraveled clustered basal bodies localized at the ciliary root in the control, but only a few scattered basal bodies were found in the oviductal isthmus of *Pgr*^*Cre*^*Cdc42*^*f/f*^ mice, indicating the defective amplification of basal body (Supplementary Fig. 3b). These results were further confirmed by immunostaining of PAX8, a marker of secretory cells, and FOXJ1, another ciliated cell fate determinant by regulating basal body docking and axoneme formation in late-stage ciliogenesis [24]. Specifically, some PAX8 and FOXJ1 dual positive cells were found in the control oviductal isthmus, indicating the transition of secretory cells into ciliated cells (Fig. 2g). Reduction of the multiciliated cells in the oviductal isthmus of *Pgr*^*cre*^*Cdc42*^*f/f*^ mice could be caused by either increased ciliated cell death or deficient ciliated cell transition. TUNEL and cleaved caspase 3 staining showed a very few apoptotic cells in *Pgr*^*cre*^*Cdc42*^*f/f*^ and *Cdc42*^*f/f*^ oviduct (Supplementary Fig. 3c, d), indicating that absence of multiciliated cells in *Pgr*^*cre*^*Cdc42*^*f/f*^ mouse oviducts was not caused by cell apoptosis. Transcription factor TAp73 (p73) had been implicated in cell fate decisions, affecting the balance between the ciliated and the secretory cell population in the airway epithelium [25, 26]. There was a large part of p73 positive cells in the oviduct expressing Ac-tubulin, implying the ciliated cell fate commitment by p73 (Fig. 2h). However, the ratio of p73 positive cells was significantly reduced in the oviductal isthmus of *Pgr*^*cre*^*Cdc42*^*f/f*^ mice (Fig. 2i). All the above data indicated that reduced multiciliated cells number in the oviductal isthmus of CDC42 deficient mice resulted from the impaired multiciliogenesis regulatory network and multiciliated cells differentiation.

### CDC42 deficiency impairs the epithelial cell junctions and associates with augmented inflammation in the oviduct

According to the known important role of CDC42 on cellular junction, we detected the expression of adhesion junction protein E-cadherin and β-Catenin. The epithelial cells of the *Cdc42*^*f/f*^ mouse oviducts showed a pseudostratified columnar epithelial distribution as characterized by E-cadherin and β-Catenin (Supplementary Fig. 4a, b). However, the epithelial cells in the *Pgr*^*Cre*^*Cdc42*^*f/f*^ isthmus were disordered and stratified arrangement, whereby E-cadherin and β-Catenin mis-localized on the basal side of epithelial cells. We next detected the expression of tight junction proteins ZO-1 and Claudin-7. ZO-1 was detected as dots and whisker-like lines at the apical-lateral border of the plasma membrane, and Claudin-7 was entirely localized to the basal and lateral cytoplasmic region in the oviductal isthmus of *Cdc42*^*f/f*^ mice, while ZO-1 was diffusely distributed and Claudin-7 was weakly expressed in the oviductal isthmus of *Pgr*^*Cre*^*Cdc42*^*f/f*^ mice (Supplementary Fig. 4c, d). The expression of PAR-3, an important participant of tight junction by interacting with CDC42 [27], showed similar expression pattern with ZO-1 and was overt disrupted in the absence of CDC42. The defective tight junction in *Pgr*^*Cre*^*Cdc42*^*f/f*^ isthmus was further confirmed by transmission electron microscopy, and the absence of desmosomes was also observed in *Pgr*^*Cre*^*Cdc42*^*f/f*^ isthmus (Supplementary Fig. 4e). These results clearly indicated that oviduct CDC42 was indispensable for the epithelial junction formation.

Gene ontology (GO) enrichment analysis of up-regulated genes showed that leukocyte migration and inflammatory signaling pathways were significantly enriched, in addition to collagen metabolism pathway (Supplementary Fig. 5a, b). Quantitative PCR confirmed that the mRNA levels of inflammatory factors *Tnfa (Tumor Necrosis Factor Alpha)*, *Il6* and chemotactic factors *Ccl2* (with monocytes chemotactic activity), *Csf1* (with macrophage chemotactic activity) and *Cxcl15* (with neutrophil chemotactic activity) were significantly increased in the *Pgr*^*Cre*^*Cdc42*^*f/f*^ mice than that in the control (Supplementary Fig. 5c). Immunofluorescence assay showed that infiltration of CD45 positive leukocytes and F4/80 positive macrophages was significantly increased in the oviductal isthmus of *Pgr*^*Cre*^*Cdc42*^*f/f*^ mice than that in the control (Supplementary Fig. 5d). Previous studies have reported that CDC42 deficiency in kidney and lung epithelium is associated with tissue collagen deposition and fibrosis [22, 28]. There was abundant deposition of collagen, especially collagen I, under the epithelial cells of the oviductal isthmus in *Pgr*^*Cre*^*Cdc42*^*f/f*^ mice, which was absent in the control (Supplementary Fig. 5e). These results indicated that the loss of CDC42 in the epithelial cells of oviductal isthmus led to a pro-inflammatory and pro-fibrosis status, which may partly contribute to the impaired oviductal embryo transport.

### CDC42 controls oviductal secretory cells transition into multiciliated cells independent of its GTPase activity

**Fig. 3:**
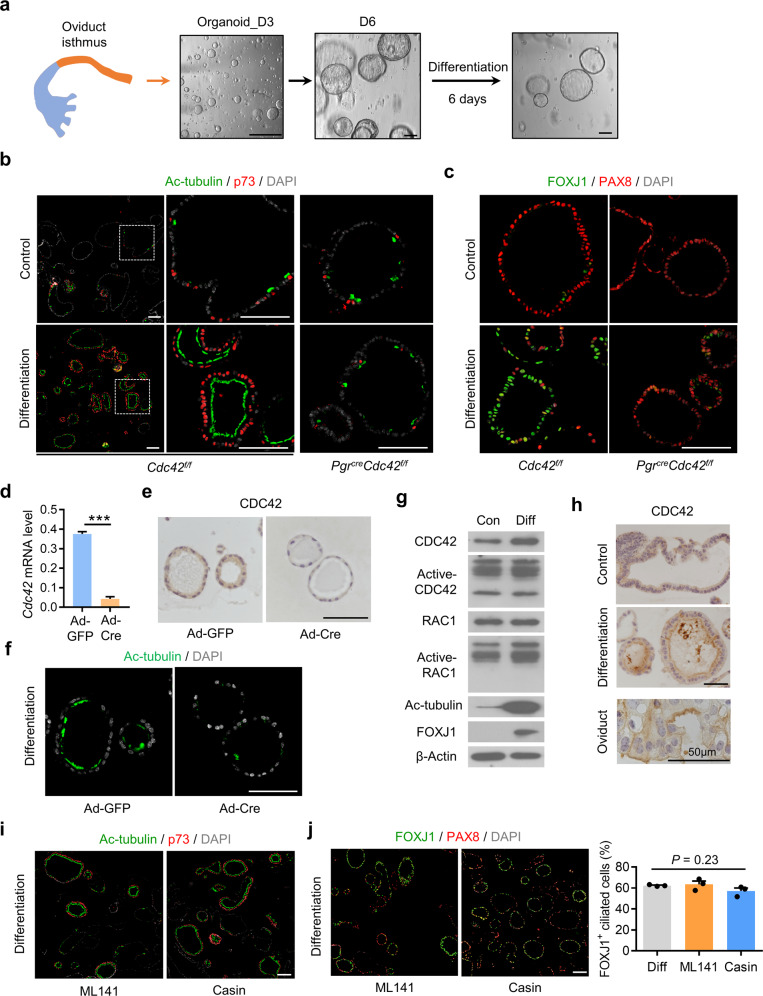
CDC42 controls oviductal secretory cells transition into multiciliated cells independent of its GTPase activity. **a** Primary epithelial cells from the isthmus were embedded in Matrigel to form oviductal organoids. (**b**, **c**) Oviductal organoids from the isthmus of *Cdc42*^*f/f*^ and *Pgr*^*cre*^*Cdc42*^*f/f*^ mouse oviducts were treated with 1 × 10^−8 ^M Estrogen (E2) and 1 μM Notch inhibitor DBZ for 6 days to induce multiciliated cells differentiation, then subjected to immunostaining of Ac-tubulin and p73 (**b**) or FOXJ1 and PAX8 (**c**). **d**, **e** Organoids from the *Cdc42*^*f/f*^ mouse oviducts were infected with adenovirus expressing the Cre recombinase gene (Ad-Cre), then subjected to RT-qPCR (**d**) and IHC (**e**) to detect the expression of CDC42 mRNA and protein. **f** *Cdc42*^*f/f*^ oviductal organoids infected with Ad-GFP or Ad-Cre were treated with E2 and DBZ, then subjected to immunostaining of Ac-tubulin. **g** Protein extracts of *Cdc42*^*f/f*^ oviductal organoids before and after E2 + DBZ treatment were subjected to western blot assay with indicated antibodies and CDC42-GTP or RAC1-GTP pull-down assays. **h** IHC assay showed CDC42 localization in the cytoplasm of epithelial cells and cilia of multiciliated cells in the organoids and oviducts. **i**, **j** *Cdc42*^*f/f*^ oviductal organoids were treated with E2, DBZ, and CDC42 activity inhibitors, ML141 (10 μM) or Casin (5 μM), then subjected to immunostaining of Ac-tubulin and p73 (**i**) or FOXJ1 and PAX8 (**j**). Scale bars, 100 μm. Mean ± SD. ***P < 0.001, Student’s *t* test. *P* = 0.23, ANOVA test.

To further explore the intrinsic role of CDC42 on multiciliated cells differentiation, the primary epithelial cells derived from the oviductal isthmus were embedded in Matrigel to form oviductal organoids (Fig. 3a). Although the *Cdc42* knockout epithelial cells from the *Pgr*^*cre*^*Cdc42*^*f/f*^ mice developed into organoids, the organoid formation capability was lower than the control organoids within 6 days (Supplementary Fig. 6a, b). Inhibition of Notch signal initiated the differentiation of precursor cells into ciliated cells, and supplement of Estrogen (E2) promoted the differentiation of ciliated cells in endometrial organoids and oviductal organoids [10, 29, 30]. After the treatment with E2 and Notch inhibitor Dibenzazepine (DBZ) for 6 days, the Ac-tubulin/p73 dual positive ciliated cells augmented significantly in *Cdc42*^*f/f*^ oviductal organoids, but remained sporadic in *Pgr*^*cre*^*Cdc42*^*f/f*^ oviductal organoids (Fig. 3b). This phenotype was further documented by FOXJ1 expression co-stained with PAX8, indicating CDC42 deficiency resulted in PAX8^+^ secretory failed to differentiate into FOXJ1^+^ multiciliated cells (Fig. 3c). To exclude the influence of long-term absence of CDC42 on organoid formation using *Pgr*^*cre*^*Cdc42*^*f/f*^ mice, CDC42 intact organoid was first established from *Cdc42*^*f/f*^ oviduct and then ablated by an adenovirus expressing the Cre recombinase gene (Ad-Cre) to establish *Cdc42* knockout (AdKO) oviductal organoids (Fig. 3d, e and Supplementary Fig. 6c). Consistently, the multiciliated cells were also declined remarkably in *Cdc42* AdKO oviductal organoids after induced by E2 and DBZ (Fig. 3f), indicating the cell-autonomous and essential role of CDC42 on multiciliogenesis.

We next interrogated whether the GTPase activity of CDC42 was required for the regulation of multiciliognesis. After E2 and DBZ induced ciliated cell differentiation as evidenced by the up-regulated Ac-tubulin and FOXJ1, the expression levels of CDC42 mRNA and protein increased notably, but the level of active-CDC42 did not show overt change, as well as active-RAC1 (Fig. 3g and Supplementary Fig. 6d, e). IHC assay revealed that increased expression of CDC42 localized in the cytoplasm and cilia in the differentiated organoids and oviduct tissues (Fig. 3h and Supplementary Fig. 6f). Then, two different CDC42 activity selective inhibitors (ML141 or Casin) were applied during the induced differentiation process to inhibit the level of active CDC42 (Supplementary Fig. 6g). The comparable FOXJ1 and Ac-tubulin positive ciliated cells in inhibitor-treated groups further corroborated the notion that CDC42 guided the differentiation of secretory cells into multiciliated cells independent on its GTPase activity (Fig. 3i, j).

### CDC42 regulates multiciliogenesis independent of Notch signaling

**Fig. 4:**
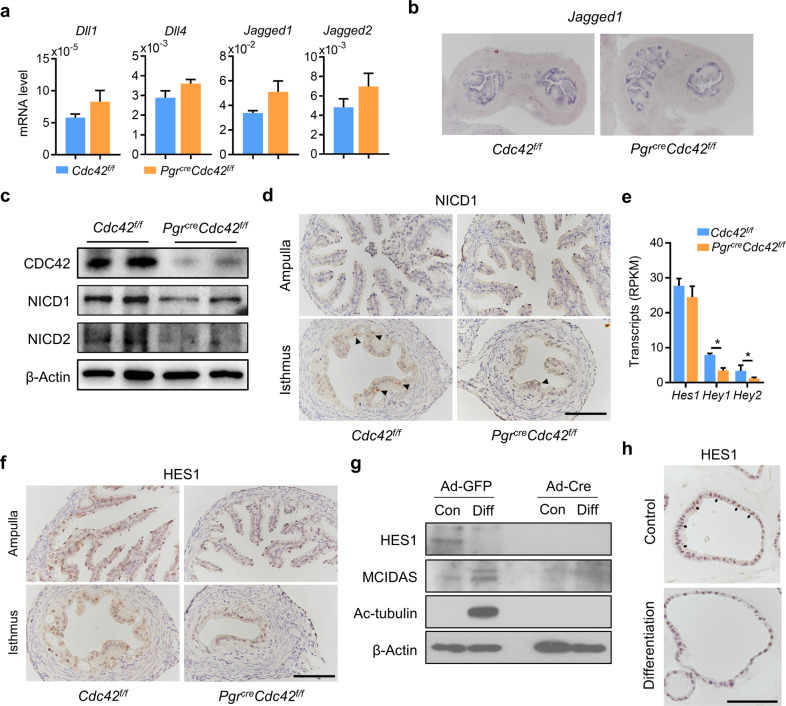
CDC42 regulates multiciliogenesis independent on Notch signaling. **a** RT-qPCR analysis of *Dll1*, *Dll4*, *Jagged1*, and *Jagged2* mRNA in *Cdc42*^*f/f*^ and *Pgr*^*cre*^*Cdc42*^*f/f*^ mouse oviducts on day 2. **b** In situ hybridization of *Jagged1* mRNA in *Cdc42*^*f/f*^ and *Pgr*^*cre*^*Cdc42*^*f/f*^ mouse oviducts on day 2. **c** Western blot analysis of CDC42, NICD1, and NICD2 protein in *Cdc42*^*f/f*^ and *Pgr*^*cre*^*Cdc42*^*f/f*^ mouse oviducts on day 2. **d** Immunostaining of NICD1 in the ampulla and isthmus of *Cdc42*^*f/f*^ and *Pgr*^*cre*^*Cdc42*^*f/f*^ mouse oviducts on day 2. **e** RNA-seq analysis of Hes1, Hey1, and Hey2 transcripts (RPKM) in *Cdc42*^*f/f*^ and *Pgr*^*cre*^*Cdc42*^*f/f*^ mouse oviducts on day 2. **f** Immunostaining of HES1 in the ampulla and isthmus of *Cdc42*^*f/f*^ and *Pgr*^*cre*^*Cdc42*^*f/f*^ mouse oviducts on day 2. **g** *Cdc42*^*f/f*^ organoids were infected with Ad-GFP and Ad-Cre, then subjected to western blot assays. **h** Immunostaining of HES1 in the organoids before and after E2 and DBZ induced differentiation. Scale bars, 100 μm. Mean ± SD. *P < 0.05, Student’s *t* test or ANOVA with Tukey’s multiple comparisons test.

Notch signaling had emerged as a consistent early event in multiciliated cells differentiation [14, 16], but the above results had showed that inhibiting the Notch signaling in the *Pgr*^*cre*^*Cdc42*^*f/f*^ oviductal organoids and *Cdc42* AdKO organoids could not induce multiciliated cells differentiation, indicating the Notch signaling may not be responsible for impaired multiciliated cells differentiation in the absence of CDC42. Accordingly, we first detected the expression of Notch ligands in oviduct tissues, and quantitative PCR results showed that the mRNA levels of Notch ligand *Dll1*, *Dll4*, *Jagged1*, and *Jagged2* in *Pgr*^*cre*^*Cdc42*^*f/f*^ mouse oviducts were slightly higher than that in control without statistical difference (Fig. 4a). Among them, *Jagged1* manifested comparable expression and localization in epithelial cells of *Cdc42*^*f/f*^ and *Pgr*^*cre*^*Cdc42*^*f/f*^ oviductal isthmus (Fig. 4b). After binding with Notch ligands, the nucleus translocation of Notch intracellular domain (NICD) initiated the expression of Notch target genes, such as the HES and HEY transcription factor families [17]. We found that the expression of Notch1 intracellular domain (NICD1) and Notch1 intracellular domain (NICD2) were decreased obviously in *Pgr*^*cre*^*Cdc42*^*f/f*^ mouse oviducts than that in the control (Fig. 4c). Some NICD1 positive epithelial cells were found in the isthmus of *Cdc42*^*f/f*^ mouse oviducts, but were rarely observed in the isthmus of *Pgr*^*cre*^*Cdc42*^*f/f*^ mouse oviducts (Fig. 4d). Similarly, the expression levels of *Hes1*, *Hey1* and *Hey2*, and proportion of HES1 positive epithelial cells were relatively decreased in the *Pgr*^*cre*^*Cdc42*^*f/f*^ mouse oviducts compared with the control (Fig. 4e, f). Multicilin (MCIDAS) is considered as one of the most upstream transcriptional activators for multiciliogenesis differentiation program [31, 32]. Notch inhibition led to the activation of MCIDAS in the differentiated *Cdc42*^*f/f*^ organoids accompanied with downregulation of HES1. In the *Cdc42* AdKO organoids, even the Notch activity was low both before and after differentiation stimulus as revealed by HES1 expression level, the expression of MCIDAS was not induced (Fig. 4g, h). All the above results suggested that CDC42 controls the multiciliated cells differentiation independent of Notch signaling.

### CDC42 deficient oviductal organoids failed to respond to the differentiation induction signal

**Fig. 5:**
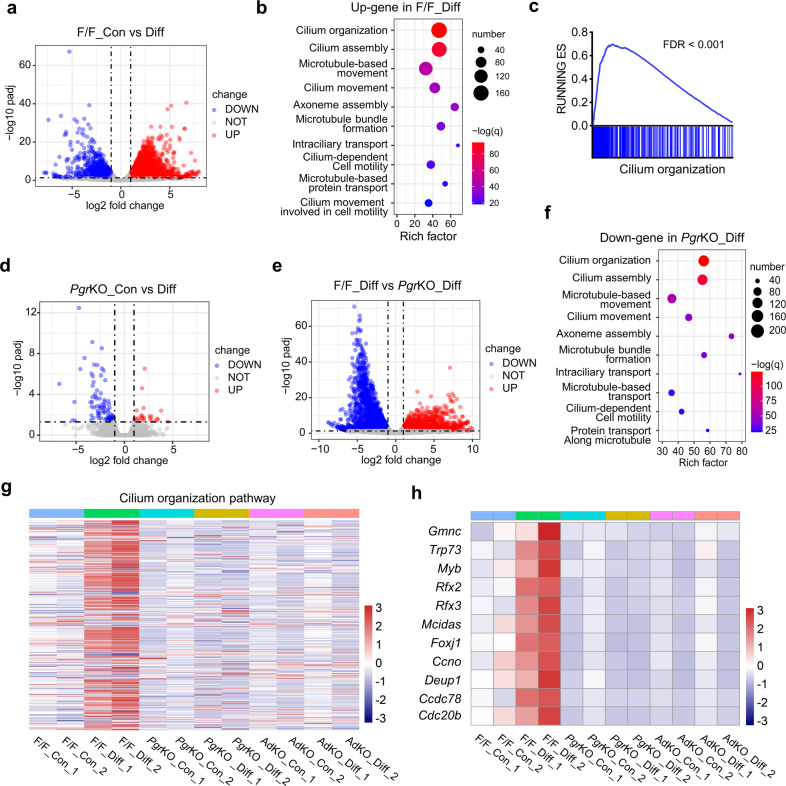
CDC42 deficient oviductal organoids failed respond to the differentiation induction signal. **a** Volcano plot of differentially expressed genes in differentiated *Cdc42*^*f/f*^ (F/F) organoids (F/F_Diff) compared with undifferentiated F/F organoids (F/F_Con). **b** Go enrichment analysis of up-regulated genes in the F/F_Diff organoids compared with F/F_Con organoids. **c** The “Cilium organization” gene set was enriched in F/F_Diff organoids according to GSEA. **d** Volcano plot of differentially expressed genes in differentiated *Pgr*KO organoids (*Pgr*KO_Diff) compared with *Pgr*KO_Con organoids. **e** Volcano plot of differentially expressed genes in *Pgr*KO_Diff organoids compared with F/F_Diff organoids. **f** Go enrichment analysis of down-regulated genes in the *Pgr*KO_Diff organoids compared with F/F_Diff organoids. **g** Heatmap based on unsupervised hierarchical clustering of cilium organization pathway (GOTerm GO:0044782) and **h** heatmap of indicated genes expression among undifferentiated F/F organoids (F/F_Con), differentiated F/F organoids (F/F_Diff), undifferentiated *Pgr*KO organoids (*Pgr*KO_Con), differentiated *Pgr*KO organoids (*Pgr*KO_Diff), undifferentiated Ad-Cre-mediated *Cdc42* knockout (AdKO) organoids (AdKO_Con), and differentiated AdKO organoids (AdKO_Diff). The expression levels in the heatmaps are provided as Z-scores with a range between −3 and 3. *N* = 2 for each group.

To further explore the mechanisms through which CDC42 regulated multiciliated cell differentiation, RNA-seq was performed to compare the transcriptomes of *Cdc42*^*f/f*^ (F/F), *Pgr*^*cre*^*Cdc42*^*f/f*^ (*Pgr*KO), and *Cdc42* AdKO organoids before and after differentiation. There was 2,023 up-regulated genes and 1159 down-regulated genes in the differentiated F/F organoids (F/F_Diff) compared with the undifferentiated F/F organoids (F/F_Con), and the up-regulated genes were significantly enriched in the pathways of cilium organization and cilia movement (Fig. 5a, b). Gene Set Enrichment Analysis (GSEA) also showed an enrichment of cilium organization pathway in the F/F_Diff organoids compared with the F/F_Con organoids (Fig. 5c). However, there was only 24 up-regulated genes and 78 down-regulated genes in the transcriptomes of undifferentiated *Pgr*KO organoids (*Pgr*KO_Con) compared with the differentiated *Pgr*KO organoids (*Pgr*KO_Diff) (Fig. 5d). We next compared the transcriptomes of F/F_Diff organoids and *Pgr*KO_Diff organoids to define the role of CDC42 on the multiciliated cell differentiation. There was 1,621 up-regulated genes and 2279 down-regulated genes in the *Pgr*KO_Diff organoids compared with F/F_Diff organoids, and the down-regulated genes were significantly enriched in the pathways of cilium organization and cilia movement (Fig. 5e, f). Heatmap based on unsupervised hierarchical clustering of cilium organization pathway (GO:0044782) further showed that F/F_Con, Ad-KO_Con, AdKO_Diff, *Pgr*KO_Con, and *Pgr*KO_Diff organoids were clustered closely, but far away from F/F_Diff organoids (Fig. 5g). The multiciliogenesis differentiation program was driven by GEMC1 (encoded by *Gmnc*) and MCIDAS to activate downstream key transcription factors p73 (encoded by *Trp73*) and FOXJ1 to program multiciliated cell specification, then underwent massive centriole amplification to generate a sufficient number of basal bodies for multiciliation [33]. These molecules in the multiciliogenesis transcriptional regulatory network, including *Gmnc*, *Mcidas*, *Myb*, *Rfx2*, *Rfx3*, *Trp73*, and *Foxj1*, were up-regulated in the F/F_Diff organoids, but not in the *Pgr*KO_Diff or AdKO_Diff organoids. Similar results were found in these molecules related to basal body amplification, including *Mcidas*, *Foxj1*, *Ccno*, *Deup1*, *Ccdc78*, and *Cdc20b* (Fig. 5h). These results indicated that CDC42 was indispensable for multiciliated cell specification and basal bodies amplification in response to the differentiation signal.

### CDC42 controls multiciliogenesis through PI3K-AKT signaling

**Fig. 6:**
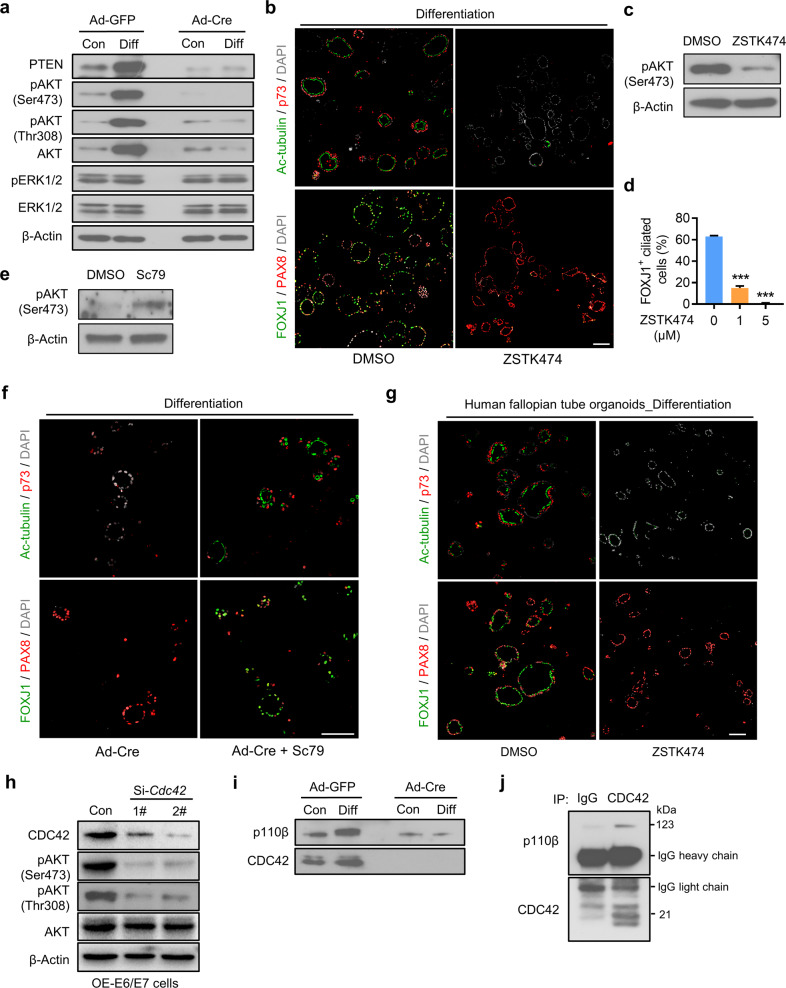
CDC42 controls multiciliogenesis dependent on PI3K-AKT signaling. **a** Western blot assays of AKT and ERK signaling activity in *Cdc42*^*f/f*^ organoids and *Cdc42* knockout organoids before and after differentiation. **b** *Cdc42*^*f/f*^ organoids were treated with E2, DBZ and PI3K inhibitor ZSTK474 (5 μM) for 6 days, then subjected to immunostaining of Ac-tubulin and p73 or FOXJ1 and PAX8. **c** Western blot assay of phosphor-AKT levels in organoids treated with ZSTK474. **d** Percentages of FOXJ1^+^ ciliated cells in differentiated oviductal organoid treated with indicated concentration of ZSTK474. **e** Western blot assay of phosphor-AKT levels in organoids treated with Sc79. **f** *Cdc42* knockout organoids treated with E2, DBZ, and AKT activator Sc79 (10 μM) showed some Ac-tubulin^+^ p73^+^ cells and FOXJ1^+^ cells. Scale bars, 100 μm. Mean ± SD. ***P < 0.001, ANOVA with Tukey’s multiple comparisons test. **g** Human fallopian tube organoids were treated with E2, DBZ, and PI3K inhibitor ZSTK474 (5 μM) for 6 days, then subjected to immunostaining of Ac-tubulin and p73 or FOXJ1 and PAX8. **h** Immortalized human fallopian tube secretory cells were transfected with si-*Cdc42* and subjected to Western blot assay. **i** Western blot assays of p110β and CDC42 in *Cdc42*^*f/f*^ organoids and *Cdc42* knockout organoids before and after differentiation. **j** Protein extracts from differentiated oviductal organoids were immunoprecipitated with anti-CDC42 and subjected to western blot assay.

As CDC42 regulated the multiciliogenesis independent of its GTPase activity and the well-known Notch pathway, we next sought to figure out the potential alternative pathway during this process. After exploring the activation status of some critical signal pathways, it was noticed that the expression level of phospho-AKT (Ser473) was increased in the *Cdc42*^*f/f*^ organoids after differentiation, but were not in the *Cdc42* AdKO organoids. However, another downstream effector of RTK pathway, phosphorylated ERK, did not change obviously. In particular, the protein level of total AKT and mRNA levels of Akt1, Akt2, and Akt3 were also increased in the *Cdc42*^*f/f*^ organoids after differentiation (Fig. 6a; Supplementary Fig. 7a). To clarify the role of PI3K-AKT signaling in multiciliogenesis, we next cultured the organoids with the PI3K inhibitor ZSTK474 during the induced differentiation process. The PI3K inhibitor-treated organoids presented an obvious decreased number of Ac-tubulin/p73 dual positive ciliated cells compared with the control group (Fig. 6b, c). Consistently, the ratio to FOXJ1^+^ ciliated cells significantly reduced along with the increased concentration of ZSTK474 (0 μM, 63 ± 1.3%; 1 μM, 15 ± 3.2%; 5 μM, 0.83 ± 0.45%, *P* < 0.001; Fig. 6d). We also found that inhibiting the WNT signaling with XAV939 led to a mild decrease of ciliated cell number, while inhibiting the ERK signaling with PD0325901 or SCH900353 had no obvious effect (Supplementary Fig. 7b). Next, we tested whether the AKT activator Sc79 could rescue the differentiation disorder in the *Cdc42* AdKO organoids. The results showed that treatment with Sc79 for 5 days indeed induced some secretory cells to differentiate into multiciliated cells in the *Cdc42* AdKO organoids (Fig. 6e, f). Finally, we isolated the primary human fallopian tube epithelial cells to construct organoids, in which the secretory cells could also differentiate into multiciliated cells after the treatment of E2 and DBZ (Supplementary Fig. 7c). Similarly, treatment with ML141 during the induced differentiation process did not inhibit the multiciliated cell differentiation, but treatment with ZSTK474 led to few Ac-tubulin/p73 dual positive cells and FOXJ1 positive multiciliated cells in the human fallopian tubal organoids (Fig. 6g).

To further confirm the role of CDC42 on the AKT signaling in the oviduct secretory cells, we knocked down *Cdc42* in the immortalized human fallopian tube secretory cells and found that the expression of phosphorylated AKT was reduced (Fig. 6h). A previous study had determined that CDC42 interacted with PI3 Kinase p110β to activate PI3K-AKT signaling [34]. Our data showed that the expression levels of p110β protein and mRNA were increased after differentiation in the control organoids, but not in the *Cdc42* AdKO organoids (Fig. 6i and Supplementary Fig. 7d). Co-immunoprecipitation assay with the CDC42 antibody confirmed the interaction between the endogenous CDC42 and p110β protein in the oviductal organoids (Fig. 6j). The above results indicated that CDC42 regulated the AKT activation at least partially through interacting with upstream p110β in the secretory cells, which was indispensable for the multiciliated cell fate determination and multiciliated cell differentiation.

### Decreased levels of CDC42 and phosphor-AKT in the fallopian tubes of women with ectopic pregnancy

**Fig. 7:**
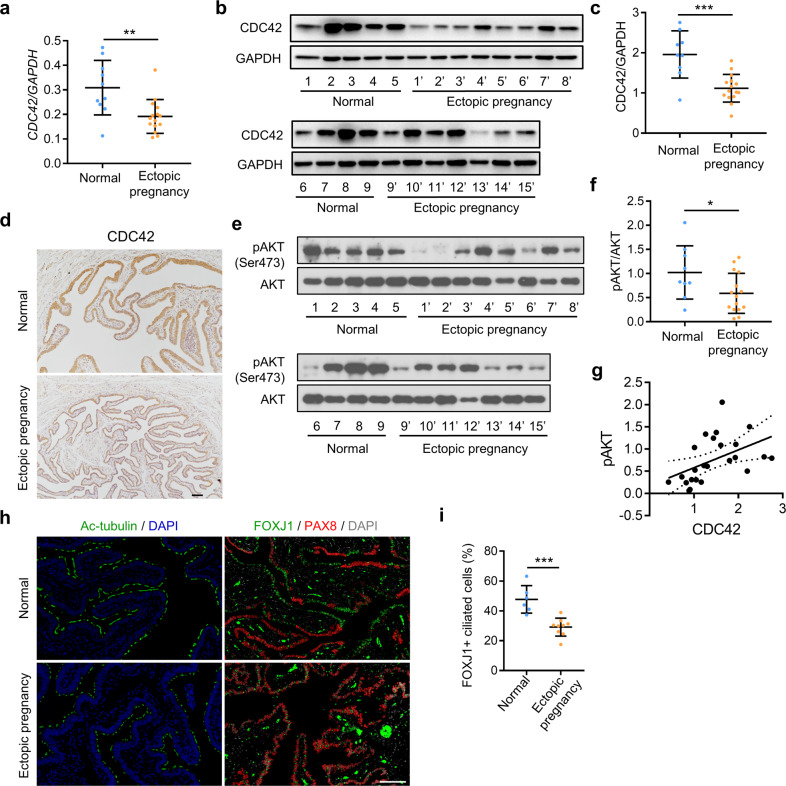
Decreased levels of oviduct CDC42 and phosphor-AKT in women with ectopic pregnancy. **a** Relative level of Cdc42 mRNA in the fallopian tubes from women with ectopic pregnancy (*n* = 15) and normal control (*n* = 10). **b**, **c** Western blot analysis of Cdc42 protein in the fallopian tubes from women with ectopic pregnancy (*n* = 15) and normal control (*n* = 9). **d** Immunostaining of CDC42 in the fallopian tubes from women with ectopic pregnancy and normal control. **e**, **f** Western blot analysis of phosphor-AKT and total AKT protein in the fallopian tubes from women with ectopic pregnancy (*n* = 15) and normal control (*n* = 9). **g** Correlational analysis of the protein levels of phosphor-AKT and CDC42 in all the fallopian tubes (*n* = 24) including women with ectopic pregnancy (*n* = 15) and normal control (*n* = 9). **h** Immunostaining of Ac-tubulin or FOXJ1 and PAX8 in the fallopian tubes from women with ectopic pregnancy and normal control. **i** Percentage of FOXJ1^+^ ciliated cells in the fallopian tubes from women with ectopic pregnancy (*n* = 9) and normal control (*n* = 6). Scale bars,100 μm. Mean ± SD. *P < 0.05, **P < 0.01, ***P < 0.001, Student’s *t* test.

Next, we detected the expression of CDC42 in the fallopian tube tissues from women with ectopic pregnancy, which may due to the defects in multiciliogenesis. Quantitative PCR and Western blot assay showed that the expression levels of CDC42 mRNA and protein were reduced in the fallopian tube tissues from women with ectopic pregnancy than that in the control group (*P* < 0.01; Fig. 7a–c). Immunostaining of CDC42 confirmed that it was expressed in the epithelial cells of human fallopian tube, while it was decreased in women with ectopic pregnancy compared with the control women (Fig. 7d). We next detected the expression level of phospho-AKT in the fallopian tube tissue, which was found to be decreased in the women with ectopic pregnancy than that in the control group (*P* < 0.05; Fig. 7e, f). In particular, we found a positive correlation between the expression levels of CDC42 and phospho-AKT in the fallopian tube tissue from women with ectopic pregnancy and control women (*P* = 0.018; Fig. 7g). We next detected the multiciliated cells distribution in the women with ectopic pregnancy showing low expression of CDC42 protein. The Ac-tubulin positive multiciliated cells were distributed in segments in the control women, but spaced along the epithelial layer in the women with ectopic pregnancy. FOXJ1 and PAX8 immunofluorescent double staining also showed the continuous distribution of ciliated cells in the control group, but abnormally isolated distribution of ciliated cells in the women with ectopic pregnancy (Fig. 7h and Supplementary Fig. 8). Statistical analysis showed that the ratio of FOXJ1 positive ciliated cells in the fallopian tube derived from women with ectopic pregnancy was reduced than that in the control women (control vs ectopic pregnancy: 47.7 ± 3.8% vs 29.1 ± 2.0%, *P* < 0.001; Fig. 7i). In addition, immunostaining of CD45 showed that the number of CD45 positive leukocytes was more in the ectopic pregnancy group than that in the control group, and there was a negative correlation between the number of CD45 positive leukocytes and the percentage of FOXJ1 positive ciliated cells in the fallopian tubes (Fig. S9a-c). We also observed a negative correlation between the number of CD45 positive leukocytes and the relative CDC42 protein levels in these samples (Fig. S9d). All the above results indicated that some women with ectopic pregnancy present the decreased expression levels of CDC42 and phospho-AKT, and reduced number of multiciliated cells in the fallopian tube, which could contribute to the occurrence of ectopic pregnancy.

## Discussion

Multiciliated cells serve basic function of many critical biological events, such as in lung and oviduct. The transcriptional regulation network dominated by Notch signaling to control multiciliated cell differentiation has made significant progress through studies in *Xenopus* and mouse, but it is still far from fully explicating the complex molecular regulation mechanism [14, 35, 36]. In this study, we provide compelling evidence of multiciliated cell fate determination directed by Cdc42 through PI3K-AKT pathway. Specially, we found that CDC42 interacts with p110β to regulate AKT signaling in multiciliogenesis, but independent on its GTPase activity. Most importantly, this essentiality of CDC42-PI3K-AKT signaling for oviduct multiciliated cell differentiation is conserved from mouse to human with aberrantly lower expression of CDC42 and phospho-AKT, and reduced ratio of multiciliated cells in both the fallopian tube tissues and organoids derived from women with ectopic pregnancy.

Most vertebrate cells have either a single non-motile primary cilium or multiple motile cilia on the cell surface. The dysfunction of motile or immotile cilia is associated with a wide range of human diseases that are known as ciliopathies [37]. A previous research has shown ciliary mutant phenotypes, including hydrocephalus and pericardial edema in the zebrafish with *Cdc42* knockdown [22]. In addition, the ciliopathies include first-order ciliopathies, which are caused by the disruption of ciliary proteins, and second-order ciliopathies which result from the disruption of non-ciliary proteins that are required for ciliary function [37]. We found that CDC42 was localized in both secretory and ciliated cells of the oviducts. As a non-ciliary protein, CDC42 protein is required for the differentiation of secretory cells into multiciliated cells. A recent study which isolated cilia from the mucociliary epithelium of *Xenopus* embryos identified 1,009 reasonably abundant ciliary proteins. CDC42 was identified in the axoneme structure of motile cilia, which is consistent with our observation that CDC42 protein localize in the multiple cilia in both oviduct tissues and oviductal organoids [38]. Therefore, we speculate that besides the cell fate transition of secretory cells into multiciliated cells, CDC42 may be involved in the ciliary elongation and intraflagellar transport which warrant further efforts.

A number of studies have shown that in different organs, multiciliogenesis shares common fate determination mechanisms. In the airways, multiciliated cells originate from p63 and SOX2-expressing progenitors [39]. In the oviduct, multiciliated cells originate from PAX8-expressing secretory cells [11]. Inhibition of Notch signaling leads to differentiation into ciliated cells at the expense of secretory cells, which is required to arrest the proliferation of progenitor cells and to specify their differentiation into multiciliated cells in the airway and oviduct [10, 16, 17]. We investigated the expression of Notch signaling, from the ligands, receptors to target genes and unraveled that Notch signaling was indeed inhibited in the *Pgr*^*cre*^*Cdc42*^*f/f*^ mouse oviduct. The lower Notch activity in the absence of CDC42 was consistent with our observation that the proliferation of *Cdc42* KO organoid was impaired, emphasizing the importance of Notch signal to balance the self-renewal and differentiation of oviduct organoid. Our data showed that inhibiting Notch signaling is insufficient to initiate the multiciliogenesis in CDC42 deficient secretory cells.

For the underlying mechanism of multiciliogenesis, the involvement of AKT is rare report before. We found an obvious increase of phospho-AKT, total AKT, p110β, and PTEN after multiciliated cell differentiation. This indicated the PTEN-AKT signal execute the cellular context depend functions in oviduct epithelial cells. PTEN has been proven to regulate multicilia formation and cilia disassembly, which occurred after the multiciliated cell fate determination [40]. Here, our results indicated that differentiated organoids presented with both increased PI3K-AKT activation and PTEN expression level, which can be explained by a recent discovery that physiologic or oncogenic PI3K activation increases PTEN protein level [41]. We did not observe a marked difference of the CDC42 GTPase activity upon multiciliated cell differentiation, and in return, inhibiting the CDC42 GTPase activity did not suppress multiciliated cell differentiation in the oviduct organoids. CDC42 activate PI3K-AKT signaling by isoform specific regulation of p110β through its RAS-binding domain [34], while its role in multiciliated cell differentiation in both mouse and human oviduct is previously undefined. Previous studies presented that p110β bound to CDC42 in a GTP dependent manner, but the interaction was obviously observed in dominant-negative GDP-bound CDC42 in living cells or purified recombinant GDP-loaded CDC42 in vitro, indicating the important role of constitutive interaction between p110β and CDC42 [34, 42]. Moreover, how the CDC42 influence the mRNA and protein level of AKT members during multiciliogenesis also deserved the exploration.

Inhibiting PI3K-AKT signaling by small molecular inhibitor or CDC42 knockout impeded multiciliated cell differentiation by silencing the target genes involved in multiciliogenesis transcriptional regulatory network. Activated-AKT promotes transcriptional competence by phosphorylating a wide range of downstream effectors central to the regulation of apoptosis, transcription factors, and oncogenic factors. Besides, various key epigenetic regulators have been identified as AKT substrates, including DNA methyltransferase DNMT1, histone methyltransferase EZH2, histone demethylases KDM5A and histone acetyltransferase p300/CBP, to be involved in the PI3K-AKT mediated transcriptional competence [43, 44]. Further exploring the epigenetic regulatory mechanism by which PI3K-AKT ensures the activation of transcriptional cascade of multiciliogenesis, including GEMC1, MCIDAS, MYB, TAp73, and FOXJ1, will broad our understanding of oviduct multiciliated cell differentiation.

Nonetheless, we provided evidence that CDC42 is an essential player in controlling the oviductal secretory cells differentiate into multiciliated cells and uncovered the novel regulatory role of PI3K-AKT signal besides the well-known Notch pathway inhibition, in governing multiciliogenesis in the oviduct (Supplementary Fig. 10). The remarkable relevance between the aberrantly CDC42-PI3K-AKT signal cascade and reduced ratio of multiciliated cells in the fallopian tube of women with ectopic pregnancy is of potential clinical significance by benefiting new therapeutic strategies development and pregnancy improvement.

## Materials and methods

### Human fallopian tube samples

Women who underwent surgical treatment for tube pregnancy were included as the ectopic pregnancy group (*n* = 15). The control samples of fallopian tube at mid-luteal phase were collected from women undergoing hysterectomy for benign gynecological conditions, without using any hormonal medication within 3 months (*n* = 10). All women had a regular 21–35 days menstrual cycle and did not have a history of tubal pathology. The collected tissues were rinsed several times to remove all visible blood, then were snap-frozen in liquid nitrogen for RNA and protein extraction, fixed in 4% paraformaldehyde (PFA) for histology and immunostaining assay, or digested to separate primary epithelial cells for organoid culture. Written consent was obtained from all participants attending the Department of Obstetrics and Gynecology of The Third Affiliated Hospital of Guangzhou Medical University.

### Mice and treatments

*Cdc42*^*f/f*^ mice containing loxP sites flanking exon 2 of the *Cdc42* gene were produced by homologous recombination as described previously [45]. *Pgr*^*Cre/+*^ mouse models were utilized to delete *Cdc42* in the female reproductive tract [23]. The conditional knockout females (*Pgr*^*Cre/+*^*Cdc42*^*f/f*^) and *Cdc42*^*f/f*^ control littermates were used in the experiments. Female mice at least 8 weeks old were mated with fertile WT males to induce pregnancy (vaginal plug = day 1 of pregnancy). The number of embryos from uterus and oviduct was calculated as a percentage of the total morula and blastocyst stage embryos collected. Mice that failed to recover any embryos were excluded from the statistical analysis. Mouse blood samples were collected on day 4 in the morning and serum P4 as well as E2 levels were measured by radioimmunoassay. Affi-Gel Blue Gel (Bio-Rad; 100‒200 mesh, no.153-7302) beads about the size of an eight-cell embryo were transferred into the oviduct umbrella of D1 pseudopregnant mice, and mice were sacrificed on D4 to check the localization of transferred blue beads. All mice were housed in the Animal Care Facility of Xiamen University, in accordance with the guidelines for the care and use of laboratory animals.

### Organoid cultures and assays

For mice organoid derivation, oviducts were dissected from *Pgr*^*Cre/+*^*Cdc42*^*f/f*^ and *Cdc42*^*f/f*^ mice. The isthmus was carefully separated under a microscope, minced, and digested with 0.5 mg/ml Collagenase type I (Sigma) and 0.012% (w/v) Dispase (STEMCELL Technologies) at 37 °C for 30 min, followed by incubation in TrypLE™ Express Enzyme (Thermo Fisher Scientific) for 10 min at 37 °C and inactivation with 1% FBS in DMEM (Gibco). Dispersed epithelia cells were mixed with Matrigel (BD Bioscience), seeded, and maintained in culture as described previously [46].

For human fallopian tube organoids, samples were washed with Dulbecco’s PBS (DPBS), and excessive connective and vascular tissue was removed. Tubes were opened longitudinally to expose the mucosal folds, washed with DPBS and incubated with Collagenase type I and Dispase for 45 min at 37 °C. The retrieved primary cells were seeded in 2D culture for 5-7 days before seeding in Matrigel for 3D organoid formation as described previously [10].

Media were changed every 2–3 days, and organoids were passaged (~10,000 cells/well) every 8 days. For passaging, growth medium was removed, and Matrigel was resuspended in cold Cultrex® Organoid Harvesting Solution and recovered by centrifugation at 500 × *g* for 5 min, then resuspended in 500 μl TrypLE Express Enzyme (Gibco) for 10 min at 37 °C. Cells were seeded as indicated for each experiment. For freezing, cells were resuspended in CELLSAVING™ Serum-Free Cell Freezing Medium (New Cell & Molecular Biotech, Suzhou, China), cooled, and stored in liquid nitrogen.

To induce *Cre* mediated *Cdc42* knockout in vitro, organoids from *Cdc42*^*f/f*^ mouse oviduct were digested as described above, then infected with recombinant adenovirus Ad5-CMV-Cre (Ad-Cre), purchased from Genechem (Shanghai, China). After incubating for 4 h, cells were recovered by centrifugation at 500 × *g* for 5 min, then embedded into Matrigel for organoid formation. To induce multiciliated cell differentiation, the oviductal organoids were treated with 1 × 10^−8^ M Estrogen (E2) and 1 μM Notch inhibitor Dibenzazepine (DBZ) for 6 days, with or without indicated inhibitors (ML141, Casin, ZSTK474, PD0325901 and SCH900353) or activators (Sc79). All the reagents were listed in Table S1.

### Oviductal epithelial OE-E6/E7 cell culture and siRNA treatment

Immortalized human oviductal epithelial OE-E6/E7 cells were maintained in DMEM/F12 containing 10% fetal bovine serum at 37 °C, 5% CO_2_. OE-E6/E7 cells were transfected with siRNA for Cdc42 and cultured for 48 h.

### Histology and immunostaining

Tissues were fixed in 4% paraformaldehyde (PFA) at 4 °C overnight. Organoids were fixed in 4% PFA at room temperature for 30 min and placed in Histogel (Thermo Fisher Scientific) before tissue processing and embedding. Formalin-fixed paraffin-embedded tissue sections (5 μm) were de-paraffinized, rehydrated, and then stained with hematoxylin and eosin (H&E) or subjected to IHC and Immunofluorescence as described previously [47]. All the antibodies were listed in Table S2. Masson’s trichrome staining was performed using Masson’s trichrome kits (Beijing Leagene Biotechnology Co., Ltd, DC0033). A TUNEL Kit (Vazyme, A111-01) was used to detect apoptotic cells according to the manufacturer’s instructions.

### In situ hybridization

In situ hybridization was performed as previously described [48]. Frozen sections (10 μm) were mounted onto poly-l-lysine-coated slides and fixed in 4% PFA solution in PBS at 4 °C. In situ hybridization with isotopes, or digoxygenin (DIG)-labeled, antisense RNA probes were performed on cryosections.

### Transmission electron microscopy

Mouse oviducts were fixed with 3% glutaraldehyde, post-fixed in 1% osmium tetroxide (OsO4), and embedded in EMbed 812 (Electron Microscopy Sciences, Hatfield, PA, USA) for ultrastructural analysis under a Hitachi H-7600 transmission electron microscope (Hitachi High-Technologies America, Inc., Pleasanton, CA, USA).

### Western blot analysis

Tissues and organoids were homogenized in whole-cell lysis buffer (50 mM Tris-HCl pH 7.6, 150 mM NaCl and 1.0% NP-40) containing a protease inhibitor cocktail (Roche Life Science) and a phosphatase inhibitor cocktail (Sigma). Western blot analysis was performed as described previously [48]. All the antibodies were listed in Table S2.

### Co-IP binding assays

Co-IP assays were performed as previously described [49]. Whole-cell protein (500 μg) from organoids was used for co-IP. After overnight incubation at 4 °C with 2 μg antibody, Dynabeads Protein A (Thermo Fisher Scientific, 10002D) was incubated 3 h at 4 °C and then were washed with IP washing buffer three times. Immunoprecipitated proteins were separated by SDS-PAGE and analyzed by Western blot, using antibodies against CDC42 and p110β. The control immunoprecipitation was performed by incubating the lysates with rabbit IgG (Abmart, B30011).

### Endogenous CDC42 activity detection

CDC42-GTP (Active-CDC42) and Active-RAC1 levels in organoids before and after E2 and DBZ treatment were determined using RAC1/CDC42 Activation Magnetic Beads Pull-down Assay Kits from Merck Millipore following the instructions. In brief, 100 μg of clarified total lysates were incubated with PAK-1 PBD magnetic beads in microfuge tube at 4 °C for 1 h, and the amount of total and active CDC42 or RAC1 were detected by western blot. G-LISA Cdc42 Activation Assay Biochem Kit (Cytoskeleton, BK127) was used to measure levels of activated CDC42 in organoids treated with CDC42 activity inhibitors was determined using according to the manufacturer’s instructions.

### RNA extraction and real-time polymerase chain reaction analysis

A total of 1 μg RNA was used to synthesize cDNA. The expression levels of different genes were validated by real-time PCR analysis using the QuantStudio5 Real-Time PCR System (Applied Biosystems). All the real-time PCR experiments were repeated at least three times. The primers for qPCR were listed in Table S3.

### RNA-seq and data analysis

Total RNA was isolated from the mouse oviducts and organoids following the standard TRIzol extraction protocol. A total of 6 cDNA libraries (*Pgr*^*Cre/+*^*Cdc42*^*f/f*^ and *Cdc42*^*f/f*^ mouse oviducts with three biological replicates) were sequenced using the BGISEQ-500 system by Huada Gene Technology Co., Ltd. (Shenzhen, China) to generate raw reads. A total of 10 cDNA libraries from organoids were generated using V8 RNA-seq Library Prep Kit (Vazyme, NR605) according to the manufacturer’s instructions, then sequenced using Illumina Nova seq platform (PE150) by Novogene Bioinformatics Technology Co., Ltd (Beijing, China) to generate raw reads. The expression levels of genes were presented as reads per kilobase per million by Tophat2 and visualized by R.

### Statistical analysis

Each experiment was repeated at least three times. All values are expressed as the mean ± SD. No statistical methods were used to predetermine the sample size. Mice were randomly allocated to experimental groups. No blinding method was used for animal studies. There was no animal exclusion criteria. The variance was similar between the groups that were being statistically compared. Two-tailed unpaired Student’s *t* tests were used for comparisons of two groups. ANOVA with Tukey’s multiple comparisons test was applied for experiments involving more than two groups. A *P* value < 0.05 was considered statistically significant.

## Supplementary information

Supplementary Figures and Legends

Table S1

Table S2

Table S3

Original Data File

Reproducibility checklist

## Data Availability

RNA-seq data sets generated in this study have been deposited at the Gene Expression Omnibus (GEO) database under accession number GSE185465 for Cdc42f/f and PgrCre/+Cdc42f/f mouse oviducts and GSE185323 for Cdc42 F/F, Cdc42 PgrKO, and Cdc42 AdKO oviductal organoids before and after induced differentiation, respectively.
