# Supplementary material for: CDC42 governs normal oviduct multiciliogenesis through activating AKT to ensure timely embryo transport
Source: Cell Death Dis. 2022 Sep 2;13(9):757. doi: 10.1038/s41419-022-05184-y (PMC9440026; doi:10.1038/s41419-022-05184-y)
Supplement: Supplementary file 1 — Supplementary Figures and Legends [file 41419_2022_5184_MOESM1_ESM.docx]

**Supplementary Figures and Figure Legends**


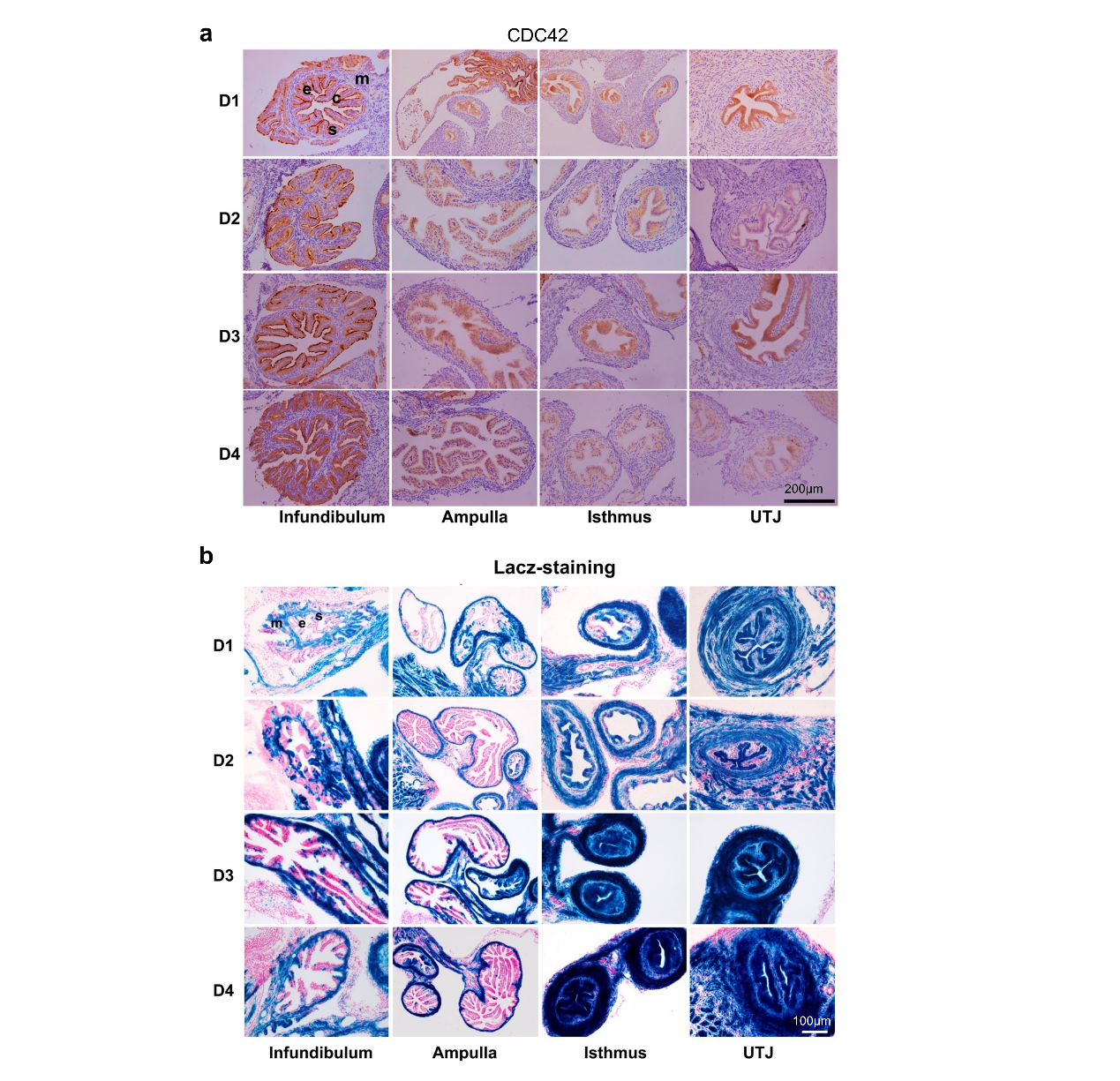


**Fig. S1.** **The expression of CDC42 and the efficiency of Pgr-Cre in the oviducts**

(a) Immunostaining of CDC42 protein in D1-4 oviducts. Scale bars, 200 μm. (b) X-gal staining of *Pgr^cre^ROSA26^Lacz^* mice oviducts on D1-4. c, cilia; e, epithelium; s, stromal; m, muscle. Scale bars, 100 μm.


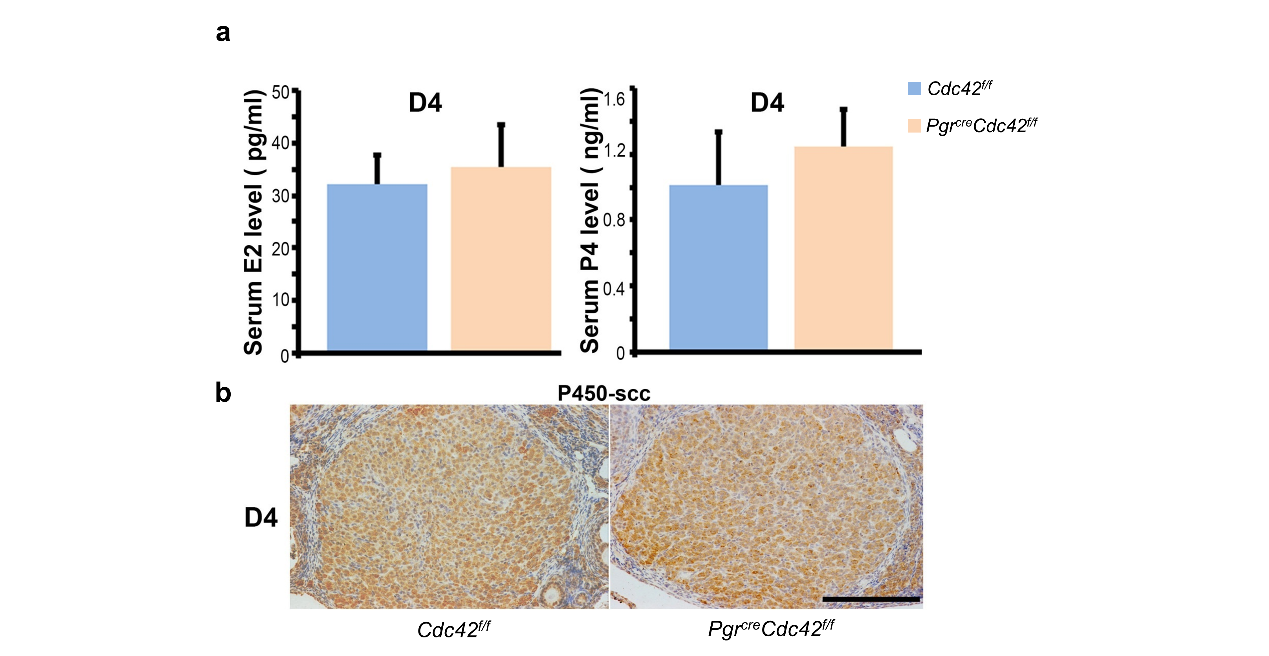


**Fig. S2.** **Apparently normal ovarian steroid hormone secretion at periimplantation in *Pgr^cre^Cdc42^f/f^* mice**

(a) Comparable serum levels of E2 and P4 in day 4 *Cdc42^f/f^* and *Pgr^cre^Cdc42^f/f^* mice. (b) Immunostaining of P450scc in day 4 *Cdc42^f/f^* and *Pgr^cre^Cdc42^f/f^* ovaries. Scale bars,100 μm. Mean ± SD. **P* < 0.05, Student’s *t* test. Scale bars, 100 μm.


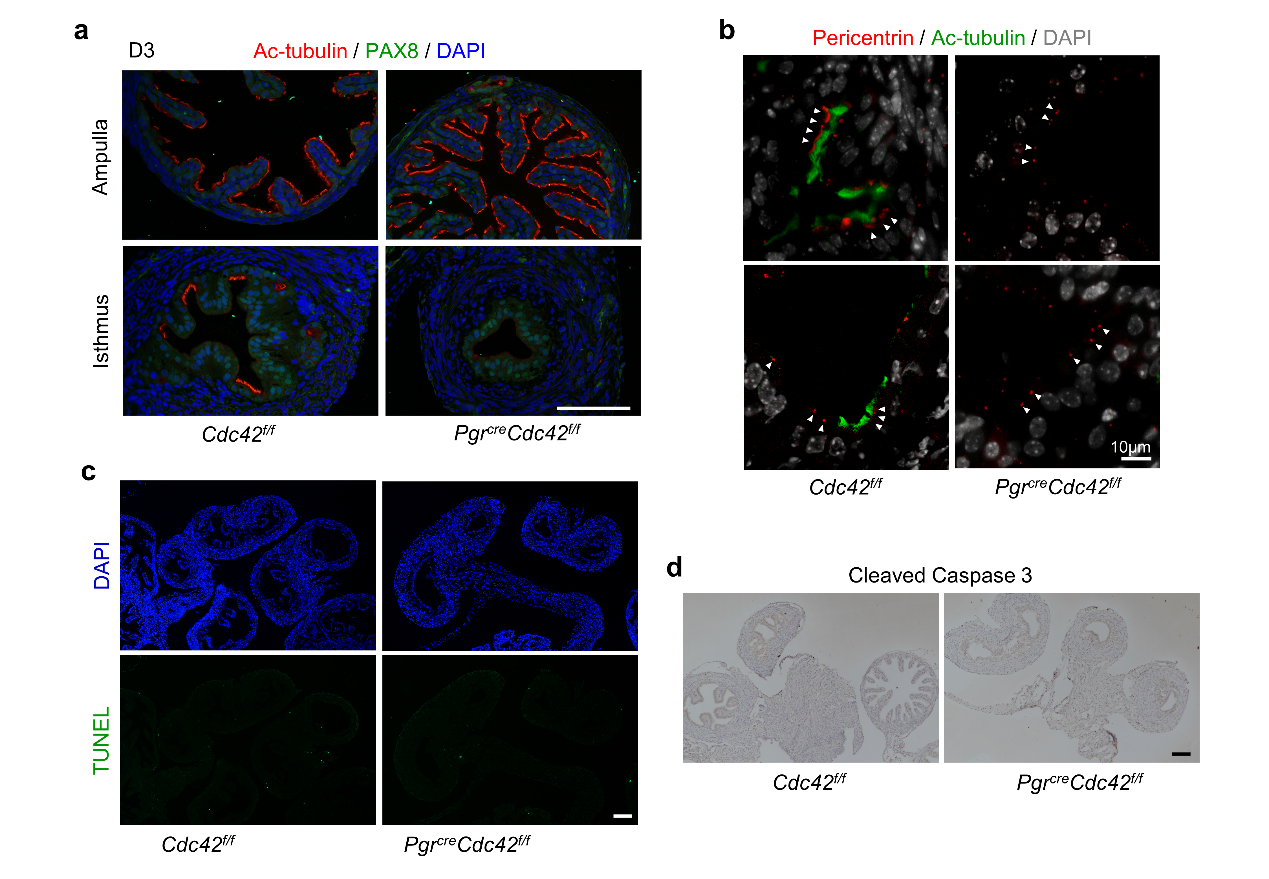


**Fig. S3.** **Pgr^cre^Cdc42^f/f^ mice showed reduced number of ciliated cells and basal bodies, without increased number of apoptotic cells.**

(a) Dual immunostaining showed the FOXJ1^+^ ciliated cells and PAX8^+^ secretory cells in the isthmus of *Cdc42^f/f^* and *Pgr^cre^Cdc42^f/f^* pregnant mice on day 3. (b) Dual immunostaining of Pericentrin and Ac-tubulin in the isthmus of *Cdc42^f/f^* and *Pgr^cre^Cdc42^f/f^* mice. (c) TUNEL staining of the *Cdc42^f/f^* and *Pgr^cre^Cdc42^f/f^* mice oviducts. (d) Immunostaining of Cleaved Caspase 3 in the *Cdc42^f/f^* and *Pgr^cre^Cdc42^f/f^* mice oviducts. Scale bars, 100 μm.


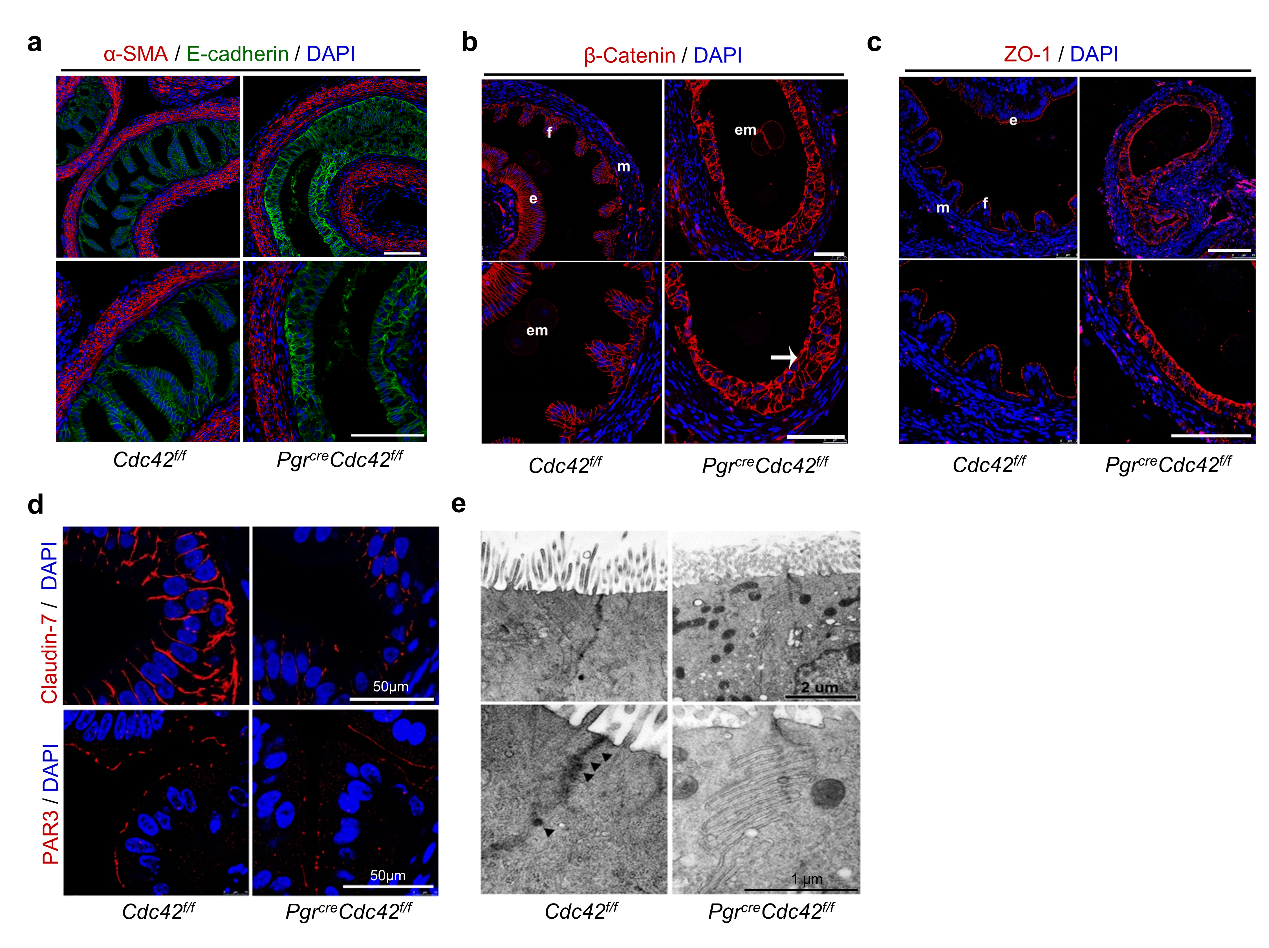


**Fig. S4. CDC42 deficiency impairs the epithelial cell junctions in the oviduct**

(a-d) Immunostaining of α-SMA and E-cadherin (a), β-catenin (b), ZO-1 (c), Claudin-7 and PAR3 (d) showed impaired adhesion junction and tight junction in the isthmus of *Pgr^cre^Cdc42^f/f^* mouse oviducts on day 2. (e) Transmission electron microscopy showed the defective tight junction and desmosomes in the isthmus of *Pgr^cre^Cdc42^f/f^* mouse oviducts on day 2. Scale bars, 100 μm.


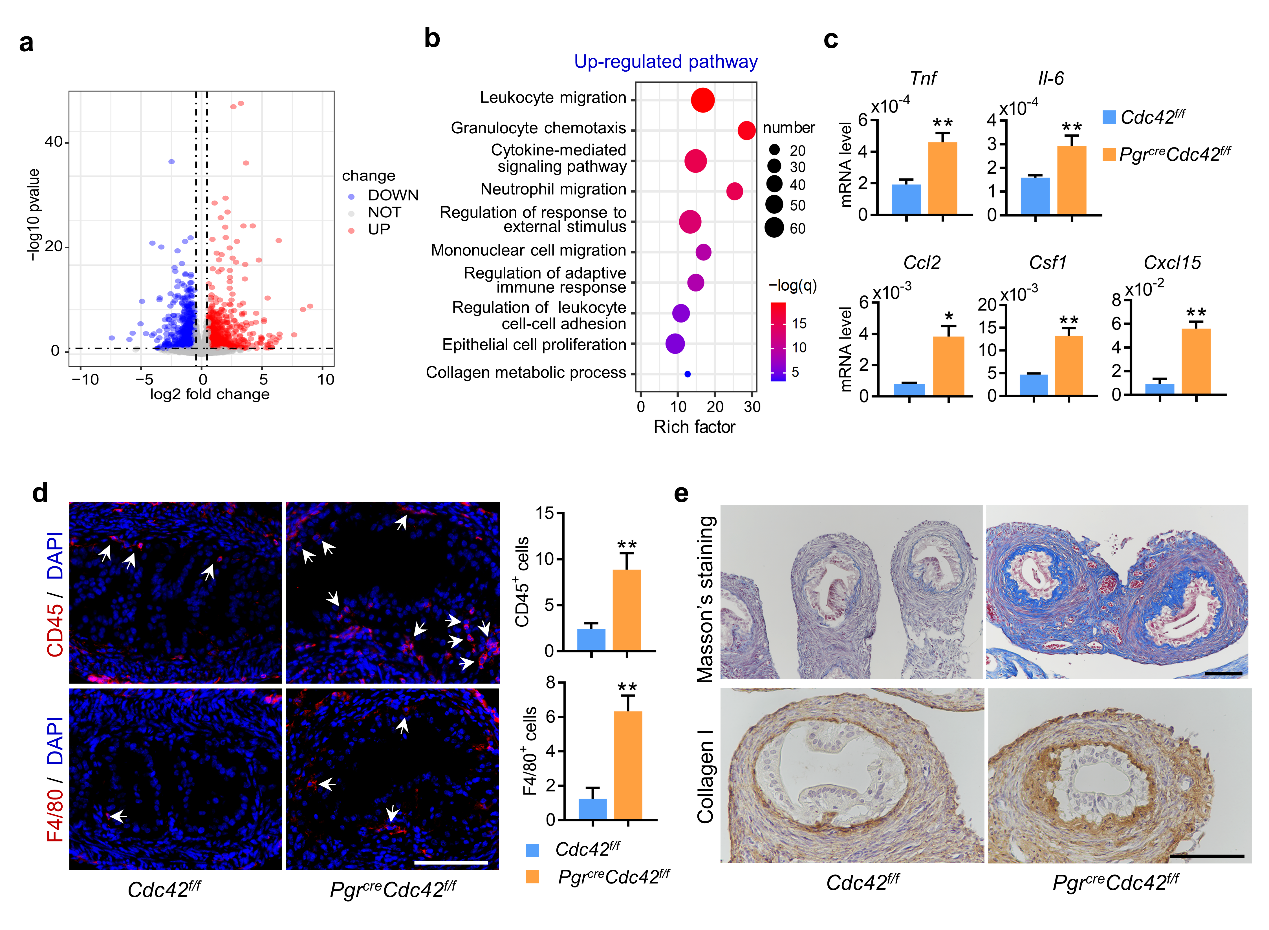


**Fig. S5.** **CDC42 deficiency is associated with augmented inflammation** **in the oviduct**

(a) Volcano plot showed significantly changed genes in the *Pgr^cre^Cdc42^f/f^* mice (n = 3) oviducts compared with that in the *Cdc42^f/f^* mice (n = 3) oviducts. (b) Go enrichment analysis of up-regulated genes in the *Pgr^cre^Cdc42^f/f^* mouse oviducts on day 2. (c) RT-qPCR analysis of Tnf, Il-6, Ccl2, Csf1 and Cxcl15 between the *Cdc42^f/f^* and *Pgr^cre^Cdc42^f/f^* mouse oviducts on day 2. (d) Immunostaining of CD45 and F4/80 showed increased number of leukocytes and macrophages in the isthmus of *Pgr^cre^Cdc42^f/f^* mouse oviducts on day 2. (e) Masson’s staining and immunostaining of Collagen I showed fibrosis in the isthmus of *Pgr^cre^Cdc42^f/f^* mouse oviducts on day 2. Arrowheads, sites of tight junction (up) and desmosomes (down). Arrows, CD45^+^ or F4/80^+^ cells. e, epithelium; f, folds; m, muscle. Scale bars, 100 μm. Mean ± SD. **P* < 0.05, ***P* < 0.01, Student’s t test.


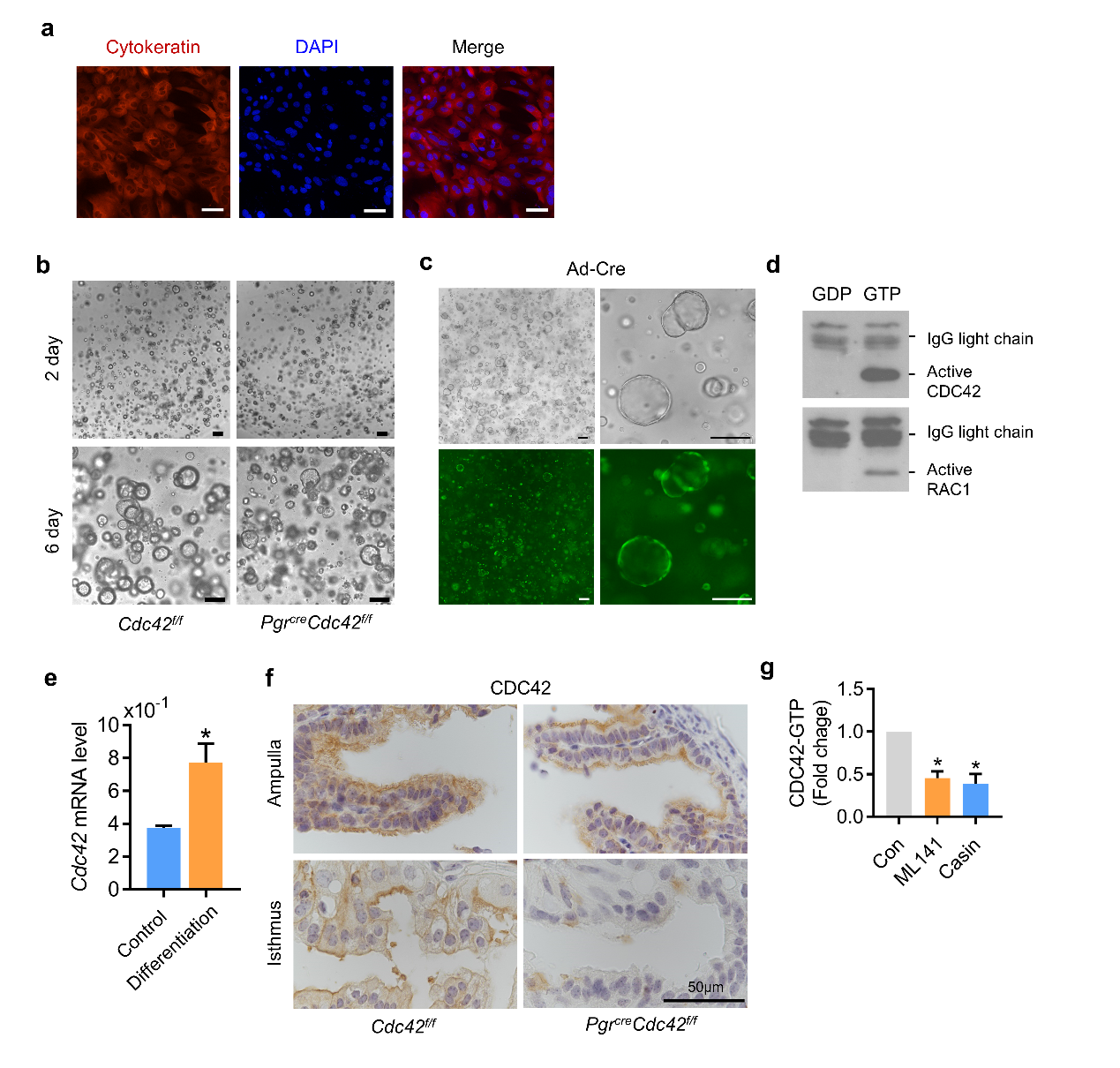


**Fig. S6.** **Construction of oviductal organoids**

(a) Immunostaining of Cytokeratin in the isolated oviduct epithelia cells. (b) Bright field images of oviduct organoids from *Cdc42^f/f^* and *Pgr^cre^Cdc42^f/f^* mice oviduct isthmus. (c) GFP fluorescence showed the infection efficiency of Ad-Cre in *Cdc42^f/f^* oviduct organoids. (d) Protein extracts from normal oviduct organoids were incubated with GDP or GTP, then subjected to western blot assay. (e) RT-qPCR assay of Cdc42 mRNA in organoids before and after E2 and DBZ induced differentiation. (f) IHC assay showed CDC42 localization in the cytoplasm of epithelial cells and cilia of multiciliated cells in the oviducts. (g) G-LISA Cdc42 Activation Assay Biochem Kit was used to measure levels of activated CDC42 in organoids treated with CDC42 activity inhibitors, ML141 (10 μM) or Casin (5 μM). Scale bars,100 μm. Mean ± SD. *P < 0.05, Student’s t test.


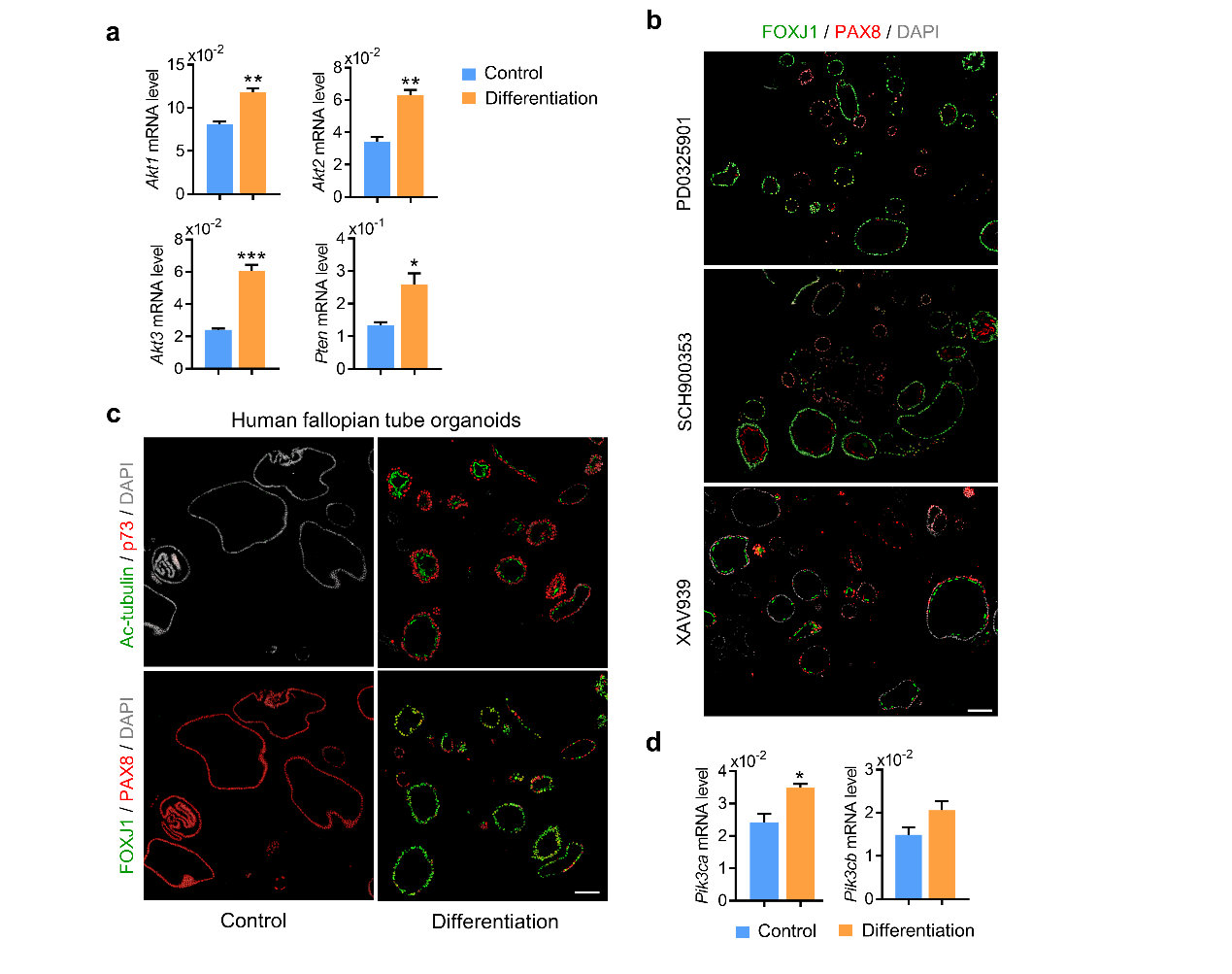


**Fig. S7.** **The role of PI3K-AKT, ERK and Wnt signaling on multiciliated cells differentiation.**

(a) RT-qPCR analysis of Akt1, Akt2, Akt3 and Pten in *Cdc42^f/f^* organoids before and after differentiation. (b) *Cdc42^f/f^* oviduct organoids were treated with 10 nM Estrogen and 1 μM Notch inhibitor DBZ, with ERK activity inhibitors PD0325901 (10 μM), SCH900353 (10 μM) or XAV939 (10 μM), then subjected to immunostaining of FOXJ1 and PAX8. (c) Human fallopian tube organoids were treated with 10nM Estrogen and 1 μM DBZ for 6 days, then subjected to immunostaining of Ac-tubulin and p73 or FOXJ1 and PAX8. (d) RT-qPCR analysis of Pik3ca and Pik3cb in *Cdc42^f/f^* organoids before and after differentiation. Scale bars,100 μm. Mean ± SD. *P < 0.05, **P < 0.01, Student’s t test.


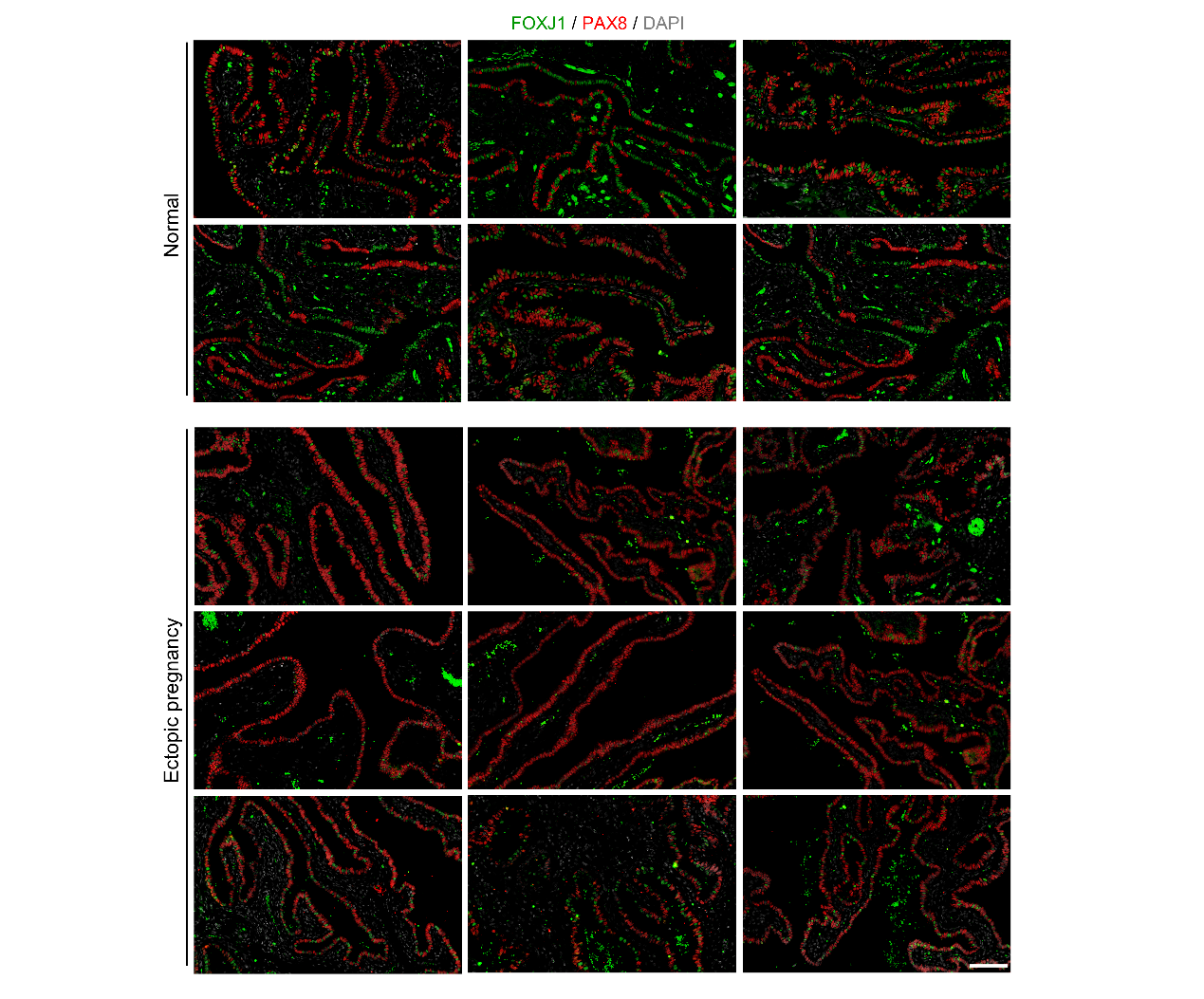


**Fig. S8.** **Reduced percentage of ciliated cells in the fallopian tubes from women with ectopic pregnancy.**

Immunostaining of FOXJ1 and PAX8 in the fallopian tubes from women with ectopic pregnancy (n = 9) and normal control (n = 6). Scale bars, 100 μm.


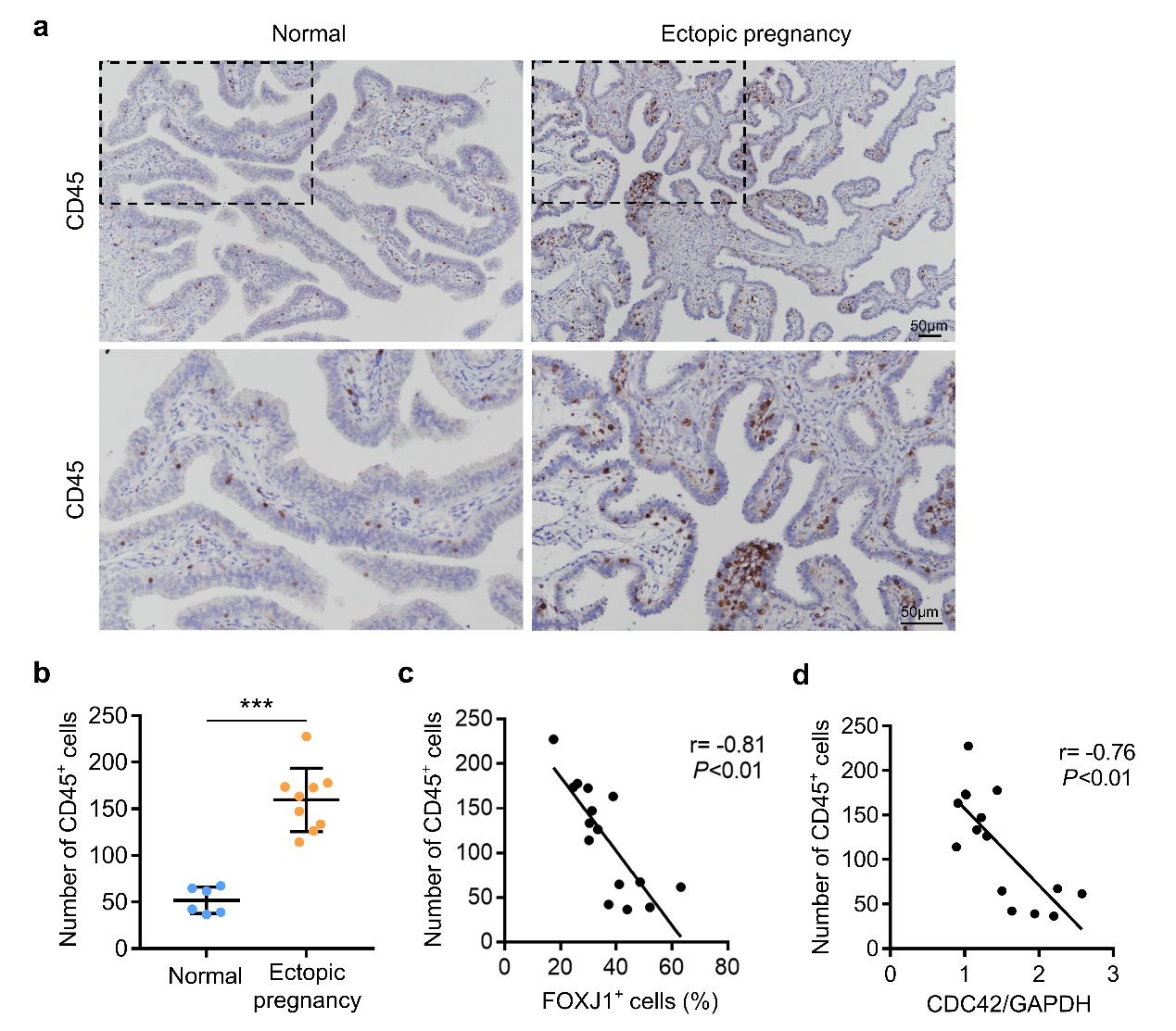


**Fig. S9. Augmented inflammation in the fallopian tubes from women with ectopic pregnancy.**

(a, b) Immunostaining of CD45 and comparison of the number of CD45^+^ in the fallopian tubes from women with ectopic pregnancy (n = 9) and normal control (n = 6). (c) Correlational analysis of the number of CD45^+^ cells and the percentage of FOXJ1^+^ ciliated cells in the fallopian tubes from women with ectopic pregnancy (n = 9) and normal control (n = 6). (d) Correlational analysis of the number of CD45^+^ cells and the relative CDC42 protein levels in the fallopian tubes from women with ectopic pregnancy (n = 9) and normal control (n = 6). Scale bars, 50 μm. Mean ± SD. ****P* < 0.001, Student’s *t* test.


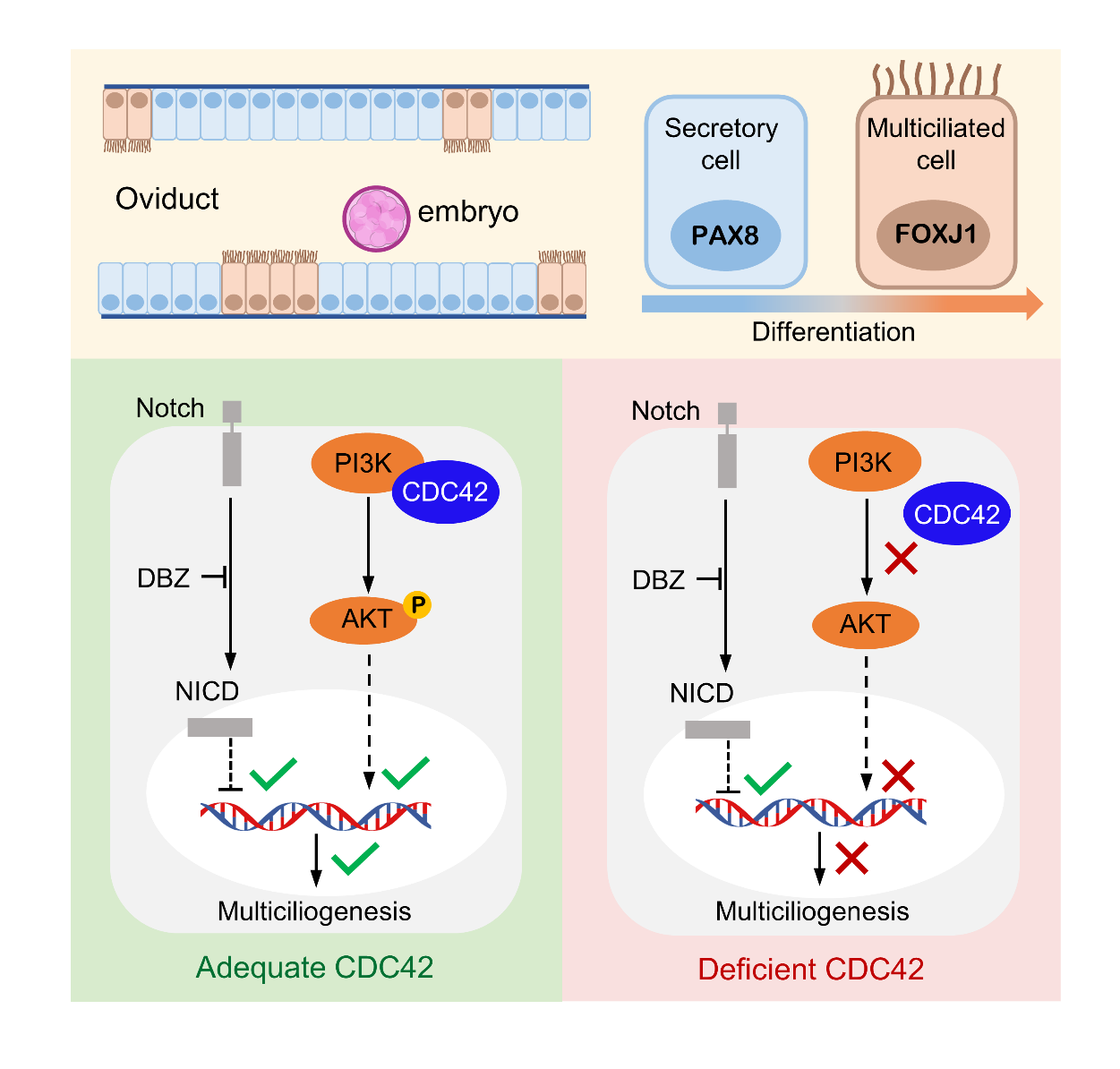


**Fig. S10. The diagram showing the critical function of CDC42-AKT signal cascade paralleled to Notch inhibition for multiciliogenesis.**

During the normal multiciliogenesis in the oviduct, besides the well-known inhibition of Notch pathway, the CDC42 regulated AKT activation is also indispensable for this process; in the deficient of CDC42, though the activity of Notch pathway is low, the multiciliogenesis is defective due to the reduced AKT activation.
