# Supplementary material for: CDC42 governs normal oviduct multiciliogenesis through activating AKT to ensure timely embryo transport
Source: Cell Death Dis. 2022 Sep 2;13(9):757. doi: 10.1038/s41419-022-05184-y (PMC9440026; doi:10.1038/s41419-022-05184-y)
Supplement: Supplementary file 5 — Original Data File [file 41419_2022_5184_MOESM5_ESM.pdf]

Figure 1

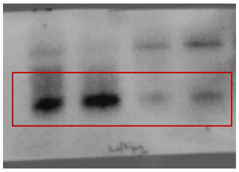

Figure 1c_CDC42

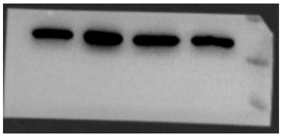

Figure 1c_β-Actin

Figure 3

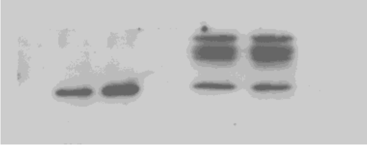

Figure 3g_ CDC42

Figure 3g_ Active-CDC42

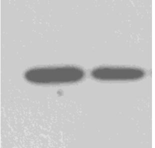

Figure 3g_ β-Actin

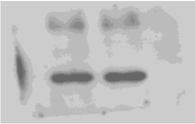

Figure 3g_RAC1

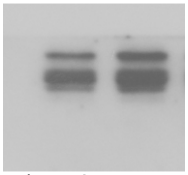

Figure 3g_ Active-RAC1

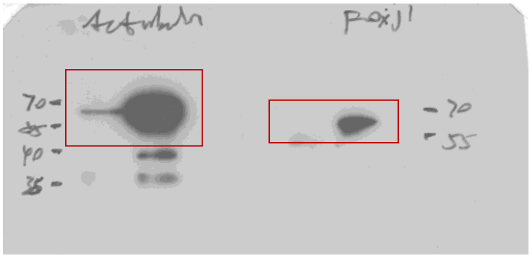

Figure 3g_ Ac-tubulin

Figure 3g_ FOXJ1

Figure 4

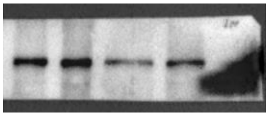

Figure 4c_NICD1

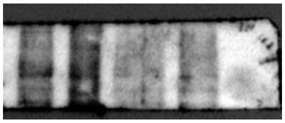

Figure 4c_NICD2

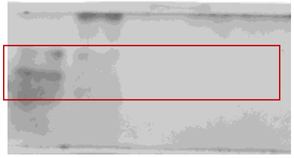

Figure 4g_HES1

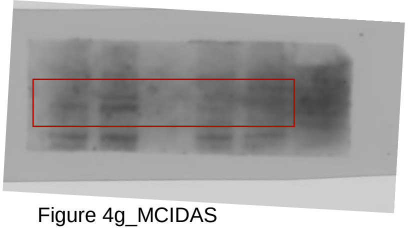

Figure 4g_MCIDAS

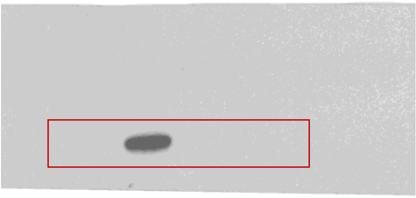

Figure 4g_Ac-tubulin

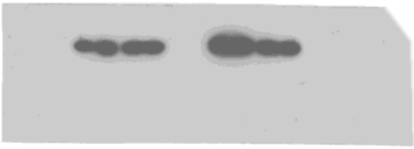

Figure 4g_β-Acitn

Figure 6

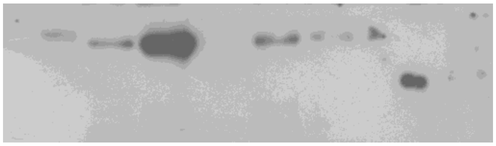

Figure 6a_AKT

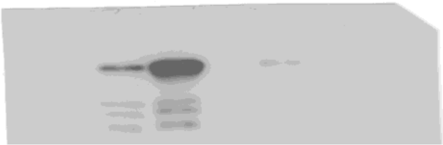

Figure 6a_pAKT(S473)

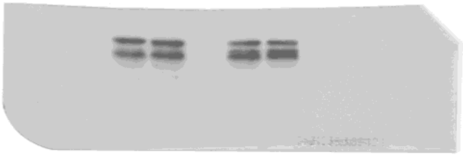

Figure 6a_ERK

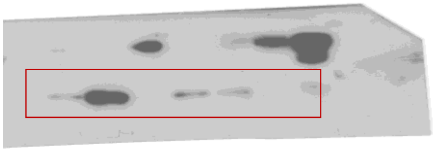

Figure 6a_pAKT(T308)

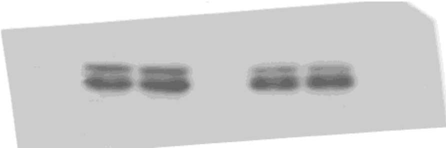

Figure 6a_pERK

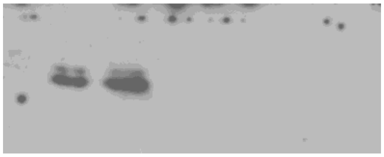

Figure 6i_CDC42

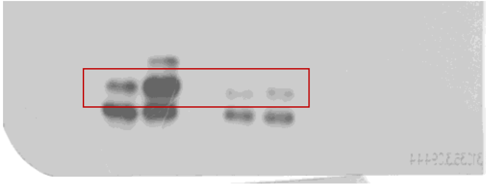

Figure 6a_PTEN

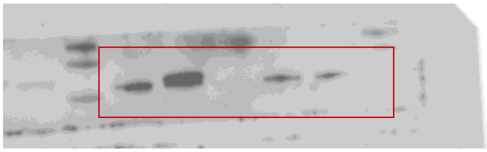

Figure 6i_p110β

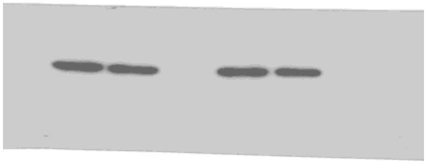

Figure 6a_β-Actin

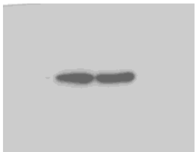

Figure 6c_β-Actin

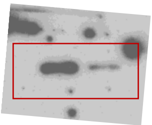

Figure 6c_pAKT

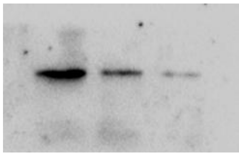

Figure 6h_CDC42

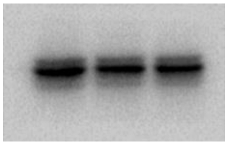

Figure 6h_AKT

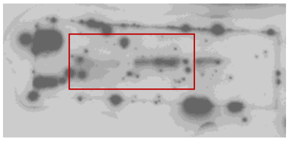

Figure 6e_pAKT

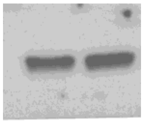

Figure 6e_β-Actin

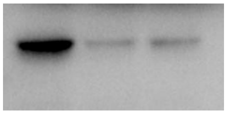

Figure 6h_pAKT(S473)

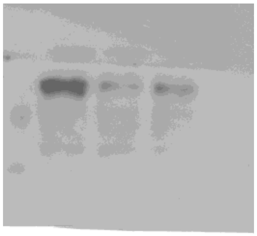

Figure 6h_pAKT(T308)

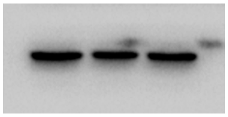

Figure 6h_β-Actin

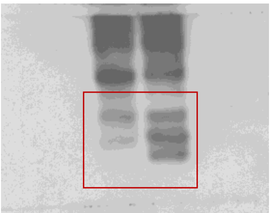

Figure 6j_CDC42

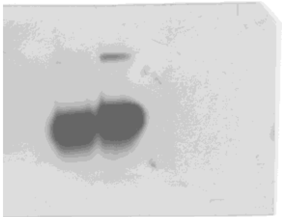

Figure 6j_p110β

Figure 7

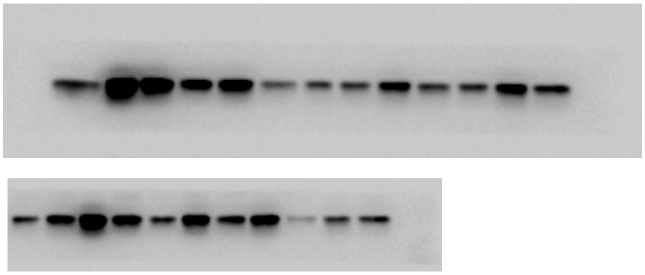

Figure 7b_CDC42

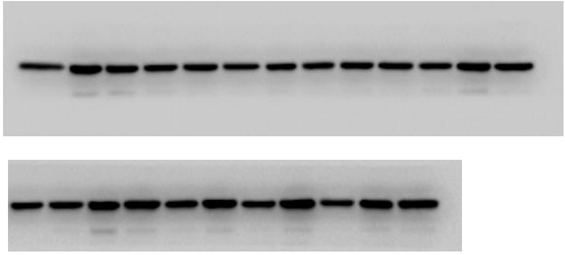

Figure 7b_GAPDH

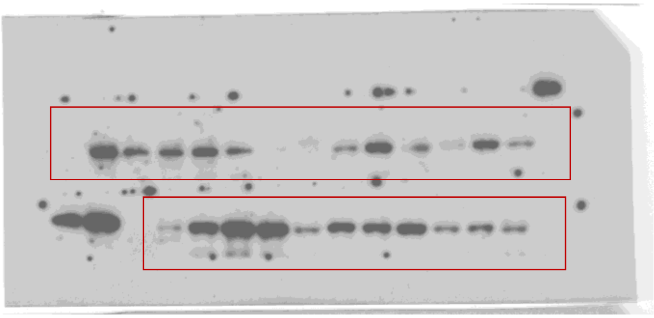

Figure 7e_pAKT

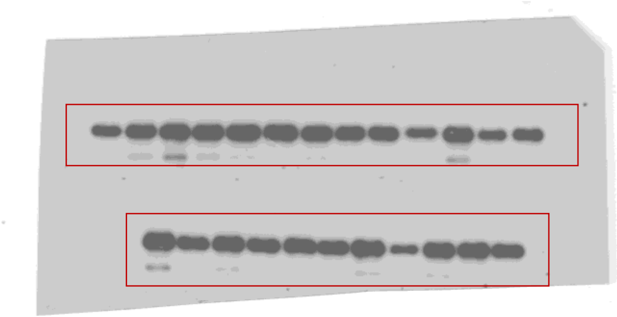

Figure 7e_AKT
